# “What are you going to do, Protest the Wind?”: Community Perceptions of Emergent and Worsening Coastal Erosion from the Remote Bering Sea Community of St. Paul, Alaska

**DOI:** 10.1007/s00267-020-01382-6

**Published:** 2020-11-07

**Authors:** Jessica Tran, Lauren M. Divine, Leanna R. Heffner

**Affiliations:** 1 Ecosystem Conservation Office, Aleut Community of St. Paul Island, 2050 Venia Minor Rd, Box 86, St. Paul, AK 99660 USA; 2 Stony Brook University, School of Marine and Atmospheric Sciences, 100 Nicolls Rd, Stony Brook, NY 11794 USA; 3 Northwest Boreal Partnership, 1227W. 9th Ave #300, Anchorage, AK 99501 USA

**Keywords:** Arctic, Climate adaptation, Climate change, Coastal management, Community perceptions, Erosion

## Abstract

The state of Alaska is experiencing increased coastal erosion due to climatic changes that threaten shoreline, infrastructure, and Alaska Native ways of life. While several Alaska Native villages have been impacted by severe erosion, additional communities face burgeoning erosion concerns. St. Paul, a remote island located in the Bering Sea, Alaska, and home to ~450 Unangan, or Aleut, residents, is experiencing relatively new erosion and associated flooding issues. This study aimed to inform St. Paul’s erosion monitoring and climate adaptation strategies by documenting community perceptions of coastal erosion as an ecological and social threat within a broader context of multiple established climate stressors. We interviewed 21 residents to answer: (1) what are the community’s perceptions of erosion on St. Paul in the context of the island’s other environmental concerns?; (2) do current perceptions of erosion affect how local governing and management entities address erosion impacts?; and (3) how does erosion relate to and impact Unangan cultural traditions and heritage? Residents identified six locations of primary concern, owing to how erosion of those areas impact their culture, subsistence practices, and sense of place. We suggest methods in which local entities can better support proactive climate adaptation and mitigation measures and utilize resources for community-driven adaption planning. By documenting perspectives in Indigenous communities on emergent climate impacts, as well as perceptions of adaptation planning and implementation, it can establish the foundation for more collaborative, culturally relevant, and successful community-driven climate adaptation planning.

## Introduction

Coastal erosion resulting from climate change impacts, such as increased storm frequency, lengthening open water periods, increased freshwater input to marine systems, and general sea level rise, represent numerous risks to coastal landscapes, infrastructure, and biological and cultural diversity (Sanò et al. 2011; IPCC 2014; Radosavljevic et al. 2016). In the Alaskan Arctic, where rates of erosion are among the highest in the world (Gibbs and Richmond 2017), many Alaska Native communities have been forced to adapt to erosion because it directly threatens their ability to remain physically and culturally connected to their ancestral lands, maintain community-oriented social networks, and continue traditional ways of life. In a 2003 study conducted by the U.S. Government Accountability Office, 184 out of 213 (86%) Alaska Native villages reported being affected by erosion and associated flooding, and this percentage is likely higher now (US GAO 2003, 2009).

Many coastal erosion studies and adaptation efforts in Alaska focus on communities experiencing extreme erosion impacts, such as areas where major infrastructure has been jeopardized or the ability to maintain traditional ways of life has been severely altered. For many of these communities, erosion has already affected residential, commercial, and public infrastructure, and led to the collapse and loss of numerous buildings (USACE Alaska District 2006a; Smith and Sattineni 2016). Some adaptation efforts in these locations include remaining in place with no action, investing in shoreline protection and reinforcement, and planning for complete relocation of a village. For example, the villages of Kivalina (Brubaker et al. 2011; Dannenberg et al. 2019), Koyukuk (USACE Alaska District 2008; Melvin et al. 2017), Newtok (Cox 2007; S. Cox, pers. comm.), and Shishmaref (USACE Alaska District 2006a, 2006b; Shen and Ristroph 2020) experienced such critical degrees of flooding and erosion that these communities are currently in the discussion, planning, or implementation stages of village relocation to less vulnerable areas. The Yup’ik village of Newtok in particular, located in western Alaska, has experienced extreme flooding and erosion that made it necessary to relocate to Mertarvik, Alaska (Bronen and Chapin 2013; Rossi 2019; Kim 2020). The Denali Commission, a federal agency who provides utilities, infrastructure, and economic support throughout Alaska, estimated that the cost to fully relocate the community will total ~$110 million (ANTHC 2018).

Although coastal erosion has been a long-standing and ever-increasing threat to some Native communities in western and northern Alaska, considerable variation exists in the rates of historical and current erosion statewide with risks to communities ranging from minimal to extreme (EPA 2017). As climate change produces variable but accelerating erosion across the Arctic, several factors are increasing impacts to Bering Sea communities that have not historically experienced coastal erosion. Warming ocean temperatures resulting in dramatic sea ice declines have destabilized historically protected shorelines and led to predictions that ice-free winters could occur in the Bering Sea by 2040 (Wang and Overland 2009; Overland et al. 2018). This loss of sea ice during the winter dramatically influences wave magnitude and may result in higher energy coastal systems (i.e., systems that experience increased erosion of sediments as opposed to deposition of sediments; Church et al. 2013; Thomson and Rogers 2014), increased storm frequency and severity (Vermaire et al. 2013), and higher rates of permafrost thaw (Raynolds et al. 2013). Erosion resulting from these environmental changes may not be initially detectable as the impacts of erosion may take months or years to become visibly noticeable (Prasad and Kumar 2014); however, observable damage may occur after an acute event such as a particularly intense storm.

To proactively respond to and prepare for impacts from these environmental changes, many communities have engaged in climate adaptation planning activities. The process of planning creates an opportunity for planners, managers, community members, and government officials to assess projected changes, threats, vulnerabilities, and opportunities from climate change impacts. In general, approaches to climate adaptation planning vary greatly. Among government agencies, such planning is often rooted in western science and is heavily reliant on quantitative data analysis, computer modeling, and geospatial data. For example, there are a variety of numerical models that estimate erosion changes, such as change from historic baselines and predicting future changes, that are increasingly applied to community adaptation planning efforts (Prasad and Kumar 2014).

In contrast, for many Indigenous communities, adaptation planning must also be rooted in Indigenous values, approaches, and traditional knowledge, alongside contributions from western science (Huntington and Watson 2012; Rosales and Chapman 2015; Tribal Adaptation Menu Team 2019). Indigenous communities hold an extensive body of knowledge based on close observations of the local environment, and this knowledge is a critical component of climate adaptation planning led by Indigenous communities (Cochran et al. 2013; Ignatowski and Rosales 2013; Manrique et al. 2018; Richards et al. 2019). Indigenous and local knowledge are invaluable parts of planning because they are well equipped to include complex social structures (Moller et al. 2004; Pearce et al. 2009; Salick and Ross 2009; Pearce et al. 2015); focus on Indigenous environments, cultures, and unique histories (Fabricius et al. 2006; Salick and Ross 2009); and connect the environmental and social changes that these communities face into appropriately framed worldviews (Berkes 2012; Ignatowski and Rosales 2013; Hosen et al. 2020).

Additionally, by understanding how a community perceives and understands climate change, it can facilitate more meaningful adaption policies. Previous literature demonstrates how differently individuals’ lived experiences and shared histories affect how adaptation options are considered, prioritized, and implemented (Marin and Berkes 2013; Herman-Mercer et al. 2016; Ambrosio-Albala and Mar Delgado-Serrano 2018; Mugambiwa and Rukema 2019). This is because, while collaborative approaches are key to instrumenting meaningful action (Callaghan et al. 2020), they can also lead to discourse with divergent opinions which, despite the obstacles, are still important in facilitating mutual support for later decisions (Curtis and Hauber 1997; Blair et al. 2014); Armitage et al. 2007). Understanding community perceptions is also invaluable because it can reveal how community members understand the organizations and institutions responsible for leading adaption efforts, thus opening the door for conversations on the structural limitations and opportunities to support community-driven adaption planning (Ali et al. 2019).

Another key feature of Indigenous-led climate adaptation planning is the continuous involvement of the community, from initial stages to implementation (Klenk and Meehan 2015; Johnson et al. 2016; Tribal Adaptation Menu Team 2019). In large part, the majority of institutions and practices around land and natural resource management tend to be made by top-down hierarchal authorities, whereas traditional Indigenous approaches value communal decision-making processes rooted in reciprocal relationships, spirituality, and respect for the land and for one another (Tribal Adaptation Menu Team 2019). Though rapid climate change indeed presents a level of urgency, climate adaptation planning efforts that are community-driven produce more effective, relevant, and fruitful strategies that are based in the knowledge of Elders, hunters and fishers, and other knowledge holders (Cochran et al. 2013; Ignatowski and Rosales 2013; Manrique et al. 2018; Richards et al. 2019; Tribal Adaptation Menu Team 2019). Community perceptions of the climate-related issues they face are important to document in the early stages of climate adaptation planning, as these can indicate emerging priorities and concerns. In addition, they can help identify potential governance, social, and economic hurdles and/or opportunities in terms of developing specific strategies (Tribal Adaptation Menu Team 2019).

Despite documented studies of how Alaska Native and Arctic Indigenous communities perceive and observe climate change (Ford et al. 2006; Carothers et al. 2014; Williams et al. 2018) and how they have long demonstrated a high degree of adaptability to climate change (Ford 2012; Pearce et al. 2015), there continue to be systemic barriers that make addressing climate change difficult. Indigenous communities face additional nonclimatic stressors like food security, healthcare insecurity, and political and social threats to self-determination, which impede their ability to implement climate adaption and mitigation policies (Corntassel 2012; Ford 2012; Loring et al. 2016; Marino 2018). As such, climate adaption planning needs to encompass more than western scientific perspectives of environmental change. It must also include addressing community issues such as poverty, health, and equity while also centering Indigenous communities’ needs as to strengthen their self-determination in decision-making (Whyte 2014; Huntington et al. 2019; Tribal Adaptation Menu Team 2019). A study focused on the community of Ruby, Alaska, argued that “adaptation to climate change is not solely about responding to the directly observable impacts of climate change…it is also about understanding and addressing the manner in which the broader political context can make communities more or less vulnerable to the impacts of climate change” (Wilson 2014). In addition, federal and state policies in place to support climate adaptation measures are rooted in western principles of capitalism, individualism, and property, which do not appropriately match the traditions, customs, worldviews, or organizational structures of Indigenous communities (Marino 2018). These obstacles place the burden of some of the most disproportionate impacts of climate change on Indigenous communities despite how they are minimally responsible for greenhouse gas emission contributions (EPA 2016; Islam and Winkel 2017).

In order to surpass these barriers, but also due to the deep knowledge and ingenuity of each community, it is valuable for communities to share and learn from one another’s efforts. Most published studies that document adaptation efforts in the Arctic tend to focus on communities that are advanced or imminently threatened. It is also notable that many of these documented efforts lack traditional knowledge inclusion in a meaningful way, as similarly found in other adaptation efforts outside of the Arctic (Boillat and Berkes 2013; Petheram et al. 2014; Carmichael et al. 2017; Ali et al. 2019; Mugambiwa and Rukema 2019). We posit that focusing on climate-related issues that are emergent—not just those that are long-standing or acute—provides a critical way to proactively address climate impacts before they become too extreme, unwieldy, or costly to sufficiently address.

St. Paul, Alaska, located in the eastern Bering Sea, is experiencing relatively new erosion and associated flooding issues compared with other areas of the state. Erosion is a comparatively new climate change related issue for the community. While the island is relatively stable and there are no immediate known threats to St. Paul’s infrastructure, specific areas have been eroding at accelerating rates in recent years and are projected to worsen in the future. The local entities responsible for erosion mitigation and adaptation measures thus far have implemented only short-term temporary solutions, such as repairing washed out roads and sand dunes by filling with dirt. The most noticeable impacts observed in vulnerable areas of the island include sand dune erosion, damage to the few gravel roads that exist for travel, and sloping and calving bluffs along shorelines. These impacts have affected important wildlife habitat, including critical rookeries (i.e., breeding grounds) and haulouts (i.e., resting areas) for northern fur seals (*Callorhinus ursinus*), and designated Important Bird Areas for seabird nesting habitats on cliffs and bluffs. Erosion is also beginning to impact the community’s access to vital subsistence resources (i.e., traditional foods that are hunted, gathered or harvested from the wild), thus increasingly threatening Unangan traditional and cultural ways of life.

This study documents community perceptions of coastal erosion as an ecological and social threat within a broader context of multiple established climate stressors by asking the following questions: (1) what are the community’s perceptions of erosion on St. Paul in the context of the island’s other environmental concerns?; (2) do the current perceptions of erosion affect how local governing and management entities address erosion impacts?; and (3) how does erosion relate to and impact Unangan cultural traditions and heritage? This study provides an opportunity to examine precautionary adaptation and mitigation steps that will facilitate avoiding an erosion-induced crisis in the future. By understanding how a community perceives and manages emergent, but worsening, erosion issues and associated impacts, valuable insights are gained as to how other remote Alaskan and broader Arctic communities in similar situations can better prepare, make proactive decisions, and adapt to a less predictable future.

## Methodologies

### Study Area

**Fig. 1:**
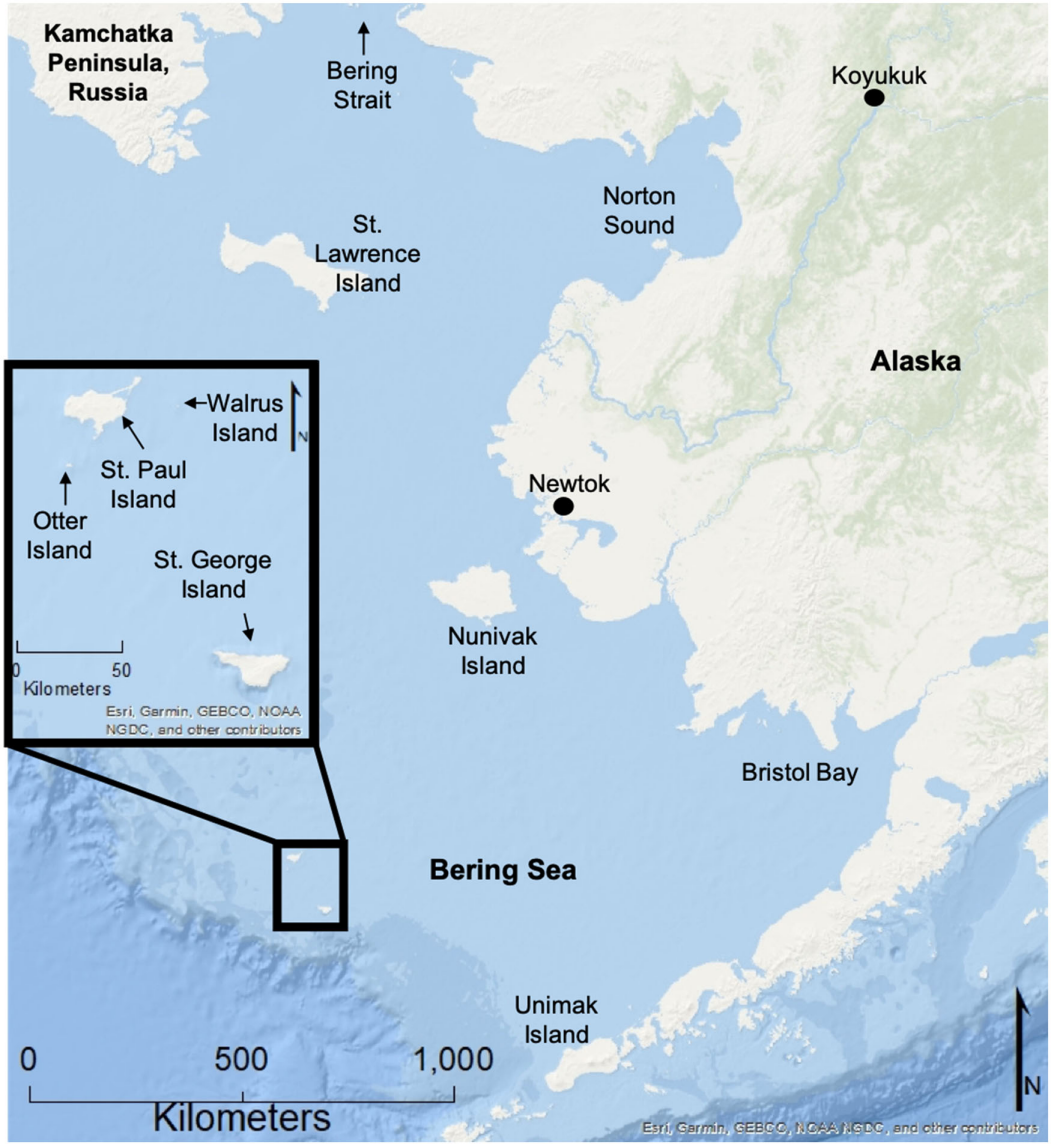
St. Paul Island’s location relative to greater Alaska and the Bering Sea. Four of the five Pribilof Islands are noted in the inset

**Fig. 2:**
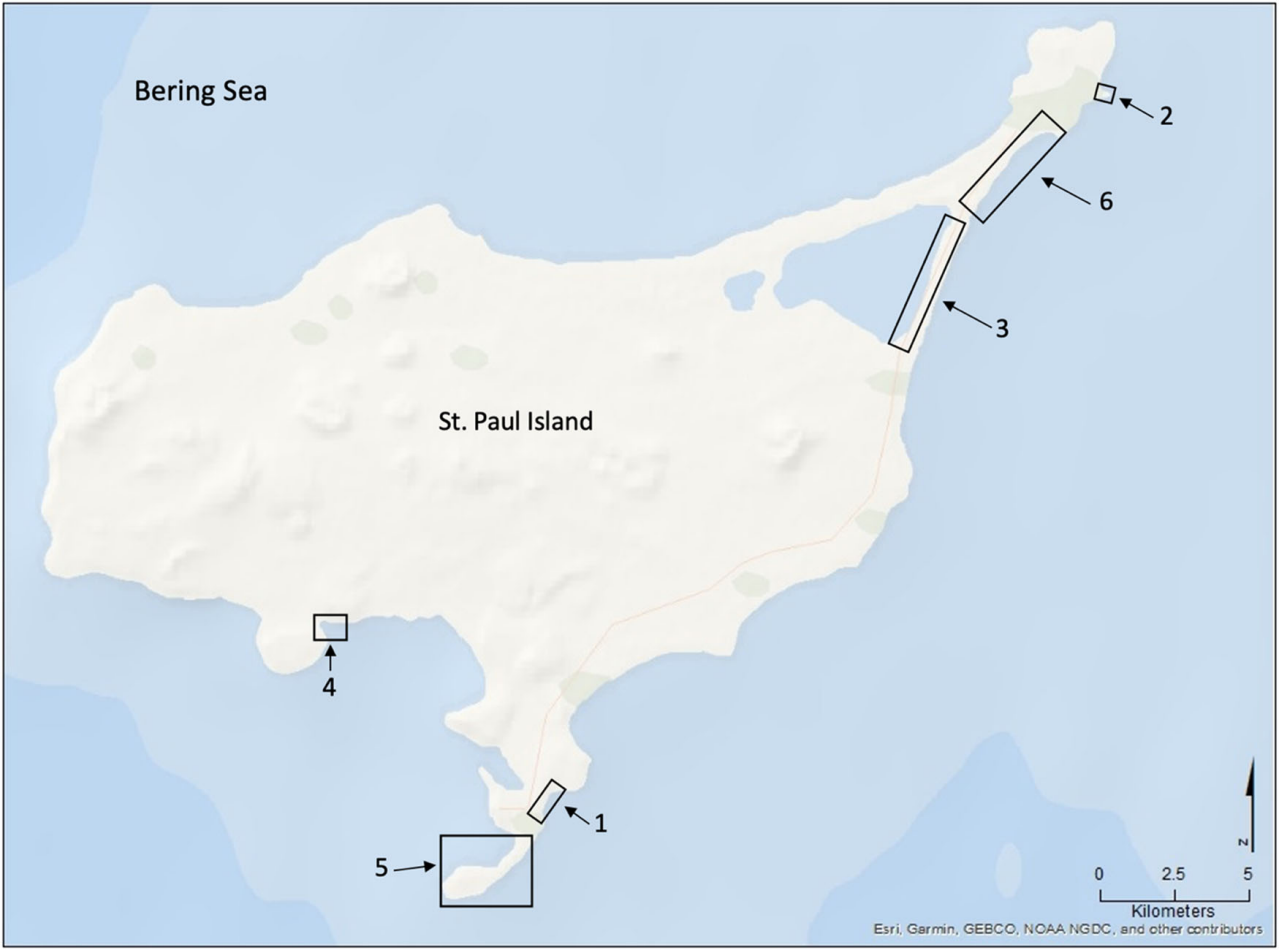
St. Paul Island, showing major features and locations of erosion concern. Location 1 is the cemetery (also known as Black Bluffs); Location 2 is Sea Lion Neck; Location 3 is the road adjacent to Big Lake; Location 4 is Zapadni haulout; Location 5 is Reef Rookery; and Location 6 is Benson Beach

St. Paul is the largest of the Pribilof Islands, a group of five volcanic islands about 400 km (~250 mi) north of the Aleutian Chain, and ~480 km (300 mi) from the mainland of Alaska (Fig. 1; Eldridge 2016). It is a highly isolated and remote island with a total land area of about 110 km^2^ (42 mi^2^). The island was geologically formed as a remainder of the Bering Land Bridge and was isolated between 14,700 and 13,500 years ago because of sea level rise during the last deglaciation (Graham et al. 2016). It is low relief with a maximum elevation of 203 m (666 ft) above sea level and contains few freshwater lakes, no springs or streams, no permafrost, and has moderately productive tundra moss vegetation (Fig. 2; Graham et al. 2016). The shorelines surrounding St. Paul range from sandy beaches of variable width, sand dunes anchored by dense beach rye and other grasses, to rocky shorelines and rugged bluffs up to ~100 m (~350 ft) but averaging 46 m (150 ft). There are currently several locations on St. Paul where erosion or accretion have been observed, but erosion has not been considered a major issue on the island within recent history. Yet, as erosion has worsened over the last decade, it is becoming an emergent issue for the community that will likely worsen over time.

St. Paul is home to ~450 residents, most of whom (~90%) are Unangax̂ (plural collective: Unangan, also known as Aleut; U.S. Census Bureau 2010). The Pribilof Islands have a unique history of permanent human inhabitance. Prehistoric inhabitants of the Pribilof Islands are descendants from the Aleutian Islands chain, with a diet based almost entirely on marine resources, including hunting marine mammals, fishing the offshore and coastal waters, foraging for fish and shellfish on the rocky reefs, and hunting birds on land and at sea. The wealth of resources on the Aleutian Islands supported dense human populations expressing a rich, strong culture (Corbett 2016). The Pribilof Islands were visited and used during prehistoric times as hunting grounds but were not permanently inhabited. After Russian contact in 1741, the Pribilof Islands were permanently inhabited for the purpose of commercial exploitation of northern fur seal pelts (McCartney 1984; Veniaminov 1984). Russian explorers enslaved Unangan hunters from Unalaska and Umnak Island to harvest pelts on the Pribilof Islands of St. George and St. Paul (Black 1983; Torrey 1983). Forced commercial harvests continued on the Pribilof Islands after the purchase of Alaska by the USA, first under the Alaska Commercial Company, then the Northern Commercial Company, and finally under the U.S. Department of Fisheries (Rubicz 2007). Commercial harvest ended in 1984 on St. Paul, and the community has since diversified the local economy, developed a municipal government, and continues to thrive as an Indigenous community.

### Characteristics of Interviewees

For this research, we aimed to include as wide of a variety of interviewees to be as representative of the St. Paul community as possible. We invited participants based on their individual experiences, skill sets, professional backgrounds and affiliations, and knowledge regarding climate change and erosion. We included participants from as wide a range of ages as possible. By including a range of interviewees, we sought to recognize how observations and memories of change have been a continuous intergenerational process, which is constantly being formed among daily conversations with fellow hunters, harvesters, other residents, and Elders (Krupnik and Jolly 2002). Additionally, the diverse backgrounds participants held provided a comprehensive set of perspectives on erosion based on local observations and individual’s interactions with each other and the environment. The following descriptions of traits and backgrounds that interviewees held represent how interviewees were targeted and asked to participate.

As the most widely encompassing trait, all interviewees were permeant residents of St. Paul. Regardless of age, gender, ethnicity, income, or any other quality, they are directly connected to erosion impacts because St. Paul is their home. The vast majority of residents have lived on the island most of their lives, if not their entire lives, and residents hold deep cultural and ancestral ties to the land. Sudden large scale or acute changes to the physical environment of St. Paul would concern and affect residents.

Subsistence hunters and harvesters on St. Paul are also particularly concerned about erosion. In addition to being key observers of climatic changes, they are directly impacted by these changes. From an Indigenous perspective, subsistence hunting and harvesting, as defined by each Indigenous community, is vital to achieving food security and central to conserving traditional ways of living. Subsistence hunters on St. Paul utilize the land year round to hunt a variety of resources including sea ducks like common eiders (*Somateria mollissima*), harlequin ducks (*Histrionicus histrionicus*), and white-winged scoters (*Melanitta fusca*); marine mammals like Steller sea lions (*Eumetopias jubatus*) and northern fur seals; and feral reindeer (*Rangifer tarandus*). Not only do individuals hunt to obtain food for themselves and their families, hunters often share their meat at community events and with others in and outside of the community who do not directly engage in hunting. This serves multiple purposes, including providing nutrition and fostering cultural connections to the natural environment. Areas of St. Paul that are significantly altered because of erosion caused by climate change will negatively affect where hunters and harvesters are able to access and obtain different cultural food sources, thus impacting the community’s overall food security. The loss of cultural foods within Unangan diets have the potential to affect individuals’ emotional and spiritual well-being (Willox et al. 2013).

Recreational participants, like ATV riders and beachcombers, comprise an additional perspective we aimed to include because, like subsistence hunters and harvesters, they spend a great deal of time in the outdoors and have observed environmental changes over time. Recreationists range in age from youth to Elder, and their individual perspectives vary depending on the activities that they participate in. Recreational participants are also concerned about the possible impacts of erosion, especially those that would prohibit future recreation in the areas of the island they currently access.

Professionally, there are three entities on St. Paul that are responsible for erosion monitoring, mitigation, and adaptation in some capacity: the Aleut Community of St. Paul Island Ecosystem Conservation Office (ECO), The City of St. Paul (The City), and the Native village corporation Tanadgusix Corporation (TDX). We interviewed residents who worked for these organizations because their specific insight and understanding of coastal erosion would allow them to have a nuanced understanding of erosion from a professional and personal standpoint.

ECO is the environmental conservation department of the federally recognized Aleut Community of St. Paul Island Tribal Government. According to their 2019–2024 Strategic Plan their mission is to, “Maintain cultural interaction with the Bering Sea environment, protect and conserve all life systems—plants, wildlife, and humans—that co-exist and are interdependent within the island’s ecosystem. Manage human activities so as not to negatively impact the natural and/or subsistence resources and other customary, traditional practices. Be respectful of and utilize both Indigenous and western approaches to environmental knowledge, wisdom, and science”. ECO has conducted erosion monitoring activities annually since 2016 by utilizing cross-shore emery rod measurements, time-lapse cameras, and distance to the edge of a dune or bluff (following Buzard et al. 2019). ECO monitors four chosen shoreline regions of St. Paul for erosion impacts and contributes its data to the State of Alaska’s Department of Geophysical and Geological Surveys (DGGS) for tracking shoreline loss across the state.

The City of St. Paul was incorporated as a second-class city in 1971 and formed a council manager form of municipal government; its limits encompass the entire island of St. Paul and three geographical miles beyond the shoreline (The City of St. Paul 2019). The municipal government assumed the responsibility and authority to provide public services which include fuel services, public safety, and the port and harbor operations, and notably in 2017 they developed a hazard mitigation plan which partially included erosion issues (Agnew::Beck Consulting 2017).

TDX is the designated Native village corporation, established under the Alaska Native Claims Settlement Act in 1971 to provide economic well-being for the Indigenous peoples who resided in the village of St. Paul, Alaska, at the time of the act’s passage by Congress, as well as their descendants (United States Code of Federal Regulations 1971). TDX’s mission is, “To develop and maintain profitable and sustainable businesses that protect and preserve the Aleut land and culture: that contribute to the successful and fulfilling lives of its shareholders through job opportunities, education opportunities, sustainable economic stability for St. Paul Island, dividends and other shareholder benefits” (TDX 2019). On St. Paul, TDX operates a wind farm and diesel power plant, fuel operations, and a tour guiding company, as well as international opportunities in technology and innovation, investment and self-determination (TDX 2019).

### Interviews and Focus Group Process and Analysis

From June to August 2019, we conducted interviews and focus groups with 21 permanent residents and tribal members, ECO employees, City of St. Paul employees, and TDX employees (Table 1). Most individuals held one or more of these roles. We also utilized our own participatory observations by living and working within in the community of St. Paul. All interviews with tribal members and permanent residents in St. Paul were conducted in-person and audio recorded.

**Table 1:** These guiding questions were applied to interviews and focus groups. In conversations with residents who held more than one background, we used a combination of questions

Interviewee Background	Interview/focus group questions
Tribal Members/ Permanent Residents	How long have you lived on St. Paul? Have you seen any changes on the island over your lifetime? What are the top three challenges of those mentioned? What do you think of erosion? Is it something you’ve seen or noticed? How would you feel or be affected if ____ eroded away? (___ is a place on St. Paul that may be threatened by erosion)
ECO Staff (tribal government)	What do you think are ECO’s top 3 environmental priorities? How do erosion patrols compare to ECO’s other responsibilities? Do you think erosion is a pressing concern right now? When did you first notice it as an issue? How do you think ECO is doing in regard to erosion monitoring?
City of St. Paul Staff (municipal government)	In regard to climate adaptation/mitigation planning, what is The City’s main concern: Flooding, storm surge, snow, other? Where are The City’s areas of primary concern for erosion issues? How does The City base its decisions on where to do erosion mitigation? How does The City work with other entities like ECO and TDX in climate adaptation? Does the local Hazard Mitigation Plan include erosion issues?
TDX Corporation Staff (village Native corporation)	Since TDX owns a majority of the land in and out of town, how does it manage erosion for the roads that lead to subsistence hunting areas? How does TDX work with other entities like the City of St. Paul and ECO in climate adaptation? Do you think that recreation (e.g., ATV riding) accelerates the erosional damage? How long would you say erosion has been an issue for TDX? What has TDX done to mitigate erosion?

Interviewees ranged from ages 8 to late 70s and were comprised of 11 males and 10 females. Of the 21 total interviewees, 7 were conducted with youth participants (under 18 years of age), 6 were adults (ages 19 to 55 years old), and 8 were Elders (ages 56 to late 70s). Individuals were asked to participate based on their permanent residency status, age (to gain a wide perspective of views), recreational interests that would provide them with knowledge regarding erosion on the island, and level of involvement in erosion monitoring or mitigation (e.g., employees that conduct monitoring activities or mitigate erosion). Initially, key informants were identified through informal conversations with ECO staff who routinely conduct traditional knowledge gathering for a range of tribal-driven projects and studies, and we employed a secondary snowball methodology where interviewees were asked to recommend other key informants during interviews. Conversations were semi-directed and derived from a list of directed questions (Table 1). We decided to utilize either a semi-structured interview or focus group based on participants’ comfort levels in responding and engaging, and conversations were intentionally casual and the interview structure was followed loosely in accordance with Huntington (1998). We encouraged interviewees to provide stories, perspectives, and observations of erosion, as well as to consider erosion issues on St. Paul in the context of other climatic changes occurring on the island.

We provided all participants with a written description of the project, contact information, and addressed any questions or concerns prior to interviews or focus groups. We obtained free, prior, and informed consent from all participants before conducting interviews or focus groups. Direct quotations were obtained from audio recordings after the voluntary waiver and consent forms were signed, and all quotations and comments have been kept anonymous. Interviews and focus groups were inductively coded (Thomas 2003) in *ATLAS.ti* (v. 8.4.4, Scientific Software Development GmbH). General inductive coding facilitated the development of codes or categories, reduction of overlap and redundancy among the categories, and organizing data into key themes that serve as the base for our interpretation of relationships. Themes were developed that articulated erosion and associated impacts with community priorities and concerns, broader climate changes, and sociocultural impacts and changes within the context of three focal questions, which we describe below.

## Results and Discussion

We sought to address three focal questions: (1) what are the community’s perceptions of erosion on St. Paul in the context of the island’s other environmental concerns? (2) Do the current perceptions of erosion affect how local governing and management entities address erosion impacts? and (3) How does erosion relate to and impact Unangan cultural traditions and heritage? Through interviews and focus groups, we identified areas of primary and secondary concern and explored coastal erosion on St. Paul in the broader context of ecological and social threats on the island. Some locations concerned residents because of their cultural significance, access to subsistence resources, or both.

We found only one theme associated with specific interviewee traits. Participants categorized as youth (age < 18) framed their perceptions of erosion as they relate primarily to recreation and enjoyment. For example, youth connected their memories of ATV riding and having picnics to how the landscape has changed. Excluding this one pattern, the lack of trends or patterns in any other categories we applied (e.g., age, sex, and hunter status) is not surprising since many respondents hold multiple roles within the community. Interviewees spoke from their backgrounds, which is a layering of any combination of the traits presented previously. For example, an individual who works for the village corporation, TDX, may also be a subsistence hunter and an ATV rider. In cases where individuals hold a professional background and experience regarding erosion-related activities, interviewees responded primarily as residents but with the expertise of their organization. Respondents answered questions about their respective organization’s erosion management practices, roles, and responsibilities, but when discussing their opinion on coastal erosion they utilized their personal and individual understanding of life on St. Paul as well as their professional experience to frame their perspectives. The descriptions of targeted interviewees’ backgrounds, characteristics, and qualities were important to define at the outset of the study, as they were used to target invitations to participate in order to gather a range of perspectives of coastal erosion on St. Paul. Despite this, backgrounds did not emerge as fundamental groupings to interpret and understand the questions of this study, thus our analysis reflects all respondents combined together.

### Locations of Primary Concern

Overall, interviewees collectively identified six locations of primary concern for erosion on St. Paul. Listed by priority or greatest concern, these locations are the community cemetery, also called Black Bluffs (47 occurrences); Sea Lion Neck (22 occurrences); the road adjacent to Big Lake (20 occurrences); Zapadni haulout (16 occurrences); Reef Rookery (10 occurrences); and Benson Beach (9 occurrences) (Fig. 2; Table 2). Each of these locations are important to the community for several reasons, either as traditionally and culturally significant sites (i.e., community cemetery), areas critical for access to subsistence hunting and/or harvesting grounds (i.e., road adjacent to Big Lake), or valuable for both subsistence access and long-enduring cultural significance to the community (i.e., Zapadni haulout).

**Table 2:** Community interview excerpts on site-specific observations of erosion for six selected sites on St. Paul Island identified as the most vulnerable to erosion impacts

Impacted site	Community interview excerpts
Cemetery (also known as Black Bluffs)	“The cemetery is falling. It’s on a cliff, so when you go out to East Landing you can see it falling and it just keeps falling.” “I’m looking at the overall landscape and how its situated with the Black Bluffs and how it would be centuries’till [it erodes]. I don’t think it’ll happen in my lifetime or yours.” “I really hope that the conversation gets brought up within the community so we can take some preventative action before we see loved ones hanging out the backside of the cliff.” “Seeing pictures from a long time ago that cliff was so out in the water, now there’s barely anything there.” “I always hear older people talking and telling me that someday the cemetery is going to fall and bodies are just going to come out. I believe it.” “It’s scary to walk behind there [Black Bluffs] because you don’t know if one [cliff] will just fall.” “It would be really devastating. People would have to move their family members. They would have to relocate the cemetery and that would be really hard for people if they had to do that.” “As a kid I always had a feeling that it was gonna go. I told my grandma that she couldn’t be buried at the top of the cemetery because I didn’t want to fish her bones out of the water. Such a random thing for such a young child to say, but I said that to her all the time.” “I wouldn’t want to relocate anything. I feel like it would be rude or disrespectful to dig up somebody’s body.” “Somebody brought up in the tribal council meeting moving the cemetery. I remember [an elder] was there and she was going, ‘You can’t move people! You can’t do this!’ and it wasn’t my idea, it was one of the tribal council members. I just said you’ve got to look at all those options because that might be the most cost effective and beneficial thing to do versus trying to throw a bunch of money and throw a bunch of rocks at it when you could get a bigger storm that washes all that out.” “The Black Bluffs right behind the cemetery has kind of fallen a lot, pretty much noticeable since the 1980s or the early 1980s you noticed a lot of erosion or stuff like that.” “Maybe the last couple of years a couple of really big cliffs have fallen off. I know those areas were where cormorants would nest, but it doesn’t seem like there’s as much of them this year than last year.” “The wave action, the spray is what’s going up there and washing that scoria loose. Even if the wave don’t hit, the spray alone hit[s] the cliffs and is washing it [away].” “I know for a fact over 50 feet [of the bluff] is gone. You see where the dugout is? When I was a kid I used to play in it because it was safe because there was more land over that way.” “We moved the cross in [from the bluff] 20 years ago, and now its [bluff edge] reaching the cross again. Last year [2018] I moved it in another 20 yards.”
Sea Lion Neck	“Come October when the storms start to hit and the high tide comes in, it starts to roll over and separate the two [Sea Lion Neck from the mainland].” “With Sea Lion Neck becoming an island you’re missing a critical hunting area for the local community of hunters who actually hunt- subsistence duck hunting and Steller sea lion hunting. That neck is a critical area for that”. “Out of all the areas, [Sea Lion Neck] has the most obvious evidence of erosion.” “Sea Lion Neck? Sea Lion Island now.” “Look how fast Sea Lion Neck eroded. It only leaves 200 feet good for hunting now. It’s no easy shot.” “I feel this will be cut off, it’s gonna happen.” “Northeast Point area kinda washed out. It seemed to wash out really fast.” “When it first started, I was teasing some guys. I said ‘Well ya know when we’re walking, we should pick up a rock and throw it down and make it pile up’.” “When its windy and rough we can’t get across because the water there is probably about two feet deep, three feet deep sometimes.” “That used to be one of the favorite hunting spots, now we can’t get to it.”
Road adjacent to Big Lake	“I definitely remember the road being a lot wider and not as scary to be driving on. I refuse to drive on that road now there because I’m scared of the truck falling off the road into Big Lake.” “It’s starting to look cut and jagged close to the road.” “The beaches, the cliffs are starting to wash out like crazy. Roads were extended way out and now the waters getting super close to where we drive now.” “We never had a problem with Big Lake because every winter it freezes over. But this past winter it hasn’t been freezing over so it’s just been washing and washing over.” “If you look around the banks on the side, that used to be all grass. You can see it where its sliding and slumping off the edges and that is something that has only happened in the last couple of years just recently with all the high winds that were having.” “You can see it on the other side, too, where the banks are just caving in. Eventually the ocean is gonna come in there.”
Zapadni haulout	“Zapadni was kind of a rude awakening, made us go ‘Woah!’” “Even out at Zapadni out towards Southwest, the wind-driven waves and the wind were starting to pull away the land by the road, so there was a couple of spots where we had to have cones up because it was digging into the road a bit.” “Zapadni was the first thing that came to mind because of the storms that we had a couple of years ago. That embankment where the seals are at, we put some rock down on it to try to save it.” “I want [the road] to go all the way around. I don’t care [how much it costs]! People love their seals and will do anything for them. The other reason I want the road to go around Zapadni is hunting and berry picking!” “The tide lines seem like they’re expanding. They’re going up higher. We had rough weather two years ago… three years ago. Zapadni road was getting threatened there, ya know? So we went in there and got the tribe [Tribal Government] to use their truck and got the tribe to move some boulders into a rock wall…” “Probably two or three feet of it is under sand already- all the rocks we put in there. Seems like the beach is getting higher.” “That used to be our killing ground right there, where Zapadni Beach is. We used to hunt right there, but now with the grass and how far the wave action is, it took the whole area out.”
Reef Rookery	“[We use that area] mainly for subsistence hunting, we get everything there. Sea lion, seal pups, kittiwakes, winter ducks, you name it.” “We used to go hunt for birds out on the little island over the peninsula out there. Not since the [water] direction has changed.” “There’s a rock pile near the cliffs there, we call that Schnaĝ. In Aleut, it means scoria rock, I think, and that one is kind of falling now too. We used to be able to sit up on top and hunt and there was an area where you can sit down and shoot, but the area has fallen.” “I worry its gonna wash out and Reef will be its own island, too.”
Benson Beach	“[Before there were] dunes and hills from the beach. That’s been blown away and taken down by the weather.” “We’re gonna have to move the road pretty soon if it keeps falling and changing.” “The dunes are going down fast, because of the wind. We have more strong winds happening now blowing it. Like Benson Road, it’s just covered all that vegetation.” “One fall I was driving by there and the waves went ‘whoosh’ and ‘whoosh’ and they went right atop my truck- that’s how bad it was.” “That area up top used to be all grass. That’s all gone.” “Hills 100 feet high, just looked like a pyramid one time, and then it’s a smaller pyramid, and now it’s just gone.”

**Fig. 3:**
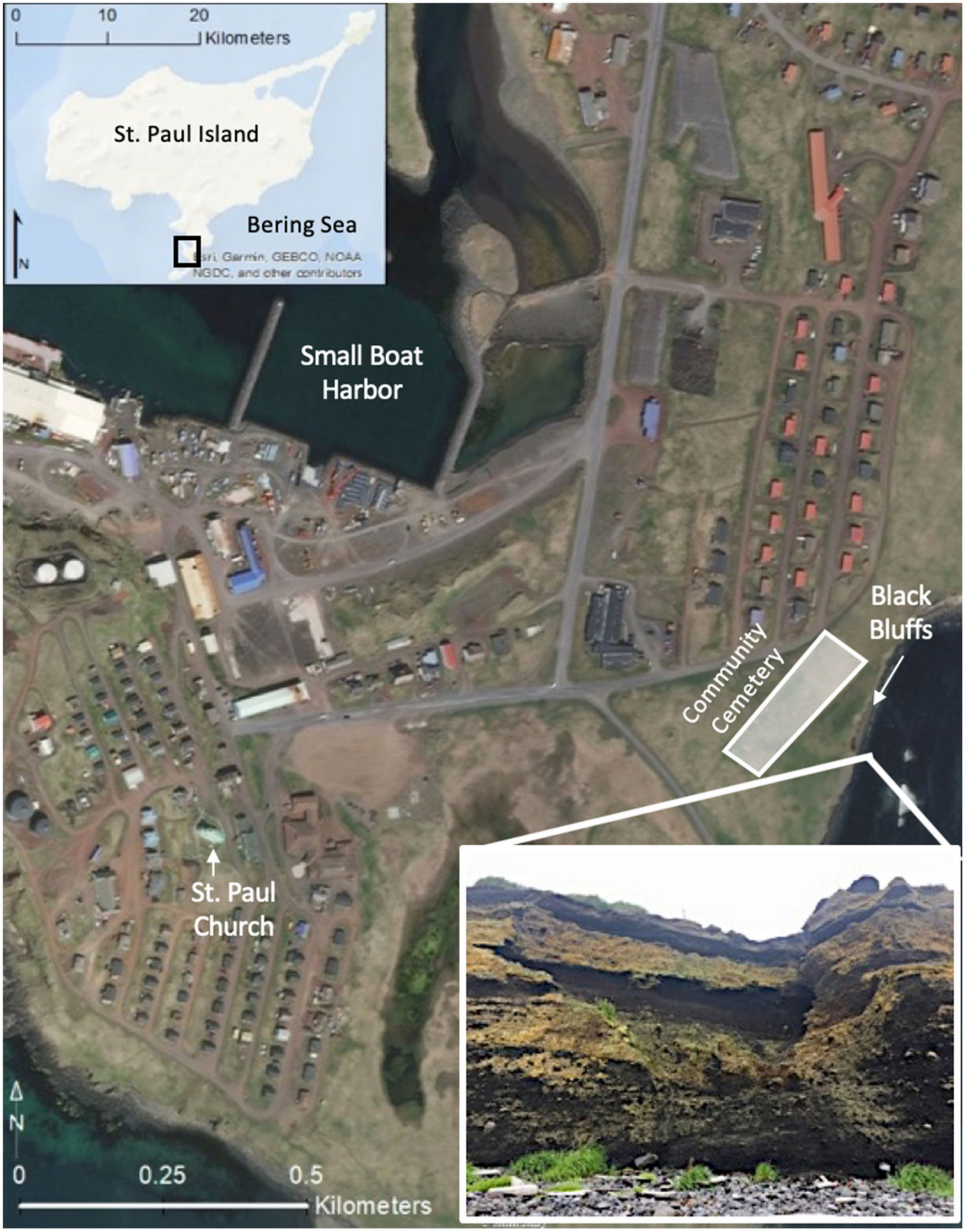
Downtown St. Paul, Alaska showing community landmarks. Black Bluffs, a presently eroding cliff face that backs up to the community cemetery, is shown in the image in the lower right corner

**Fig. 4:**
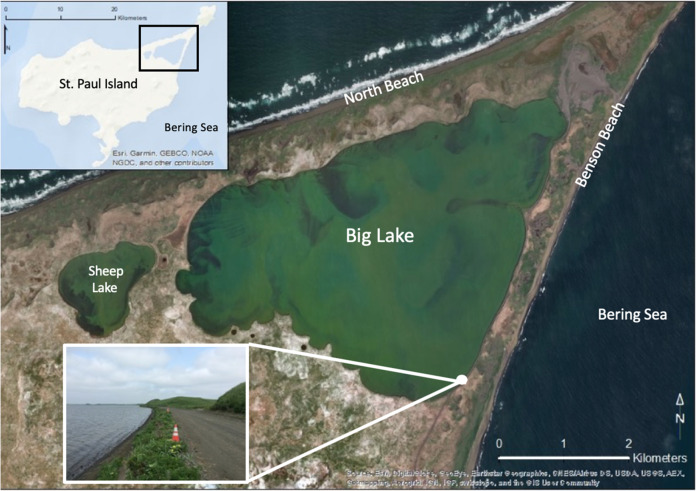
Northeast region of St. Paul Island. The image shows the road at Big Lake, with traffic cones cautioning drivers of the erosion close to the road

**Fig. 5:**
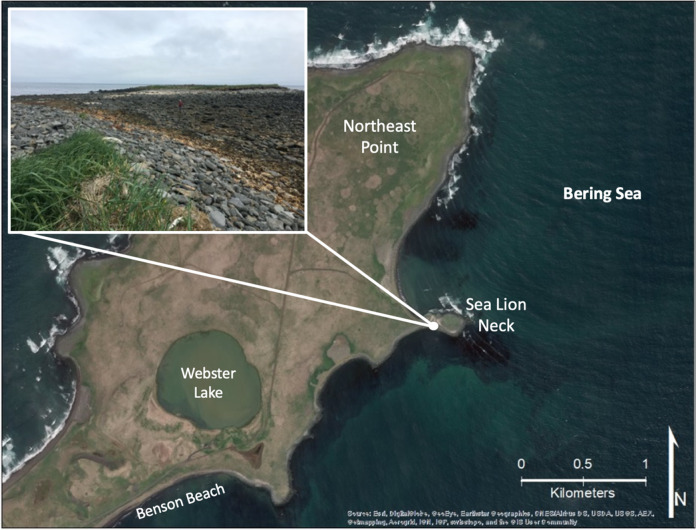
Northeast Point on St. Paul Island. Sea Lion Neck is pictured in the inset image, with the stretch of shoreline that is underwater during storms and high tides

The community cemetery is a culturally significant site, which is centrally located along an east-facing cliff known as Black Bluffs in the town of St. Paul (Figs 2 and 3). It contains the graves of almost all permanent residents since the island’s settlement during Russian occupation in 1741 and it is currently used to bury recently deceased family members (Torrey 1983). Indeed, the cemetery emerged as one of the key locations given the importance of the cemetery and its connections to history, culture, religion, and personal identity. The remaining five locations were all considered priorities because they were related in some way to subsistence or recreational uses, thus had high importance to interviewees. Reef Rookery, a northern fur seal breeding ground, is the closest rookery to the village and is a popular hunting area for ducks, seals, and seabirds and harvest area for seabird eggs (Fig. 2). Zapadni haulout, located in the southcentral portion of the island, is a resting area for nonbreeding northern fur seals during the summer (Fig. 2; P. Lestenkof, pers. comm.). The northeastern region of St. Paul encompasses the three remaining areas of concern. The road adjacent to Big Lake is ~2.5 kilometers long and is the only access route to Benson Beach and Sea Lion Neck; Benson Beach is northeast of Big Lake and faces southeast (Figs 2 and 4). Benson Beach is a popular beachcombing location and is an important monitoring location for a variety of environmental and wildlife surveys implemented by ECO. Sea Lion Neck is a small peninsula jutting from the east side of the island and is a popular sea lion hunting ground (Figs 2 and 5; Lestenkof et al. 2018). Due to increasing rates of erosion in recent years, some residents have nicknamed the peninsula “Sea Lion Island” or “Morjovi Island” because of how it separates from the mainland during storms and high tides.

**Fig. 6:**
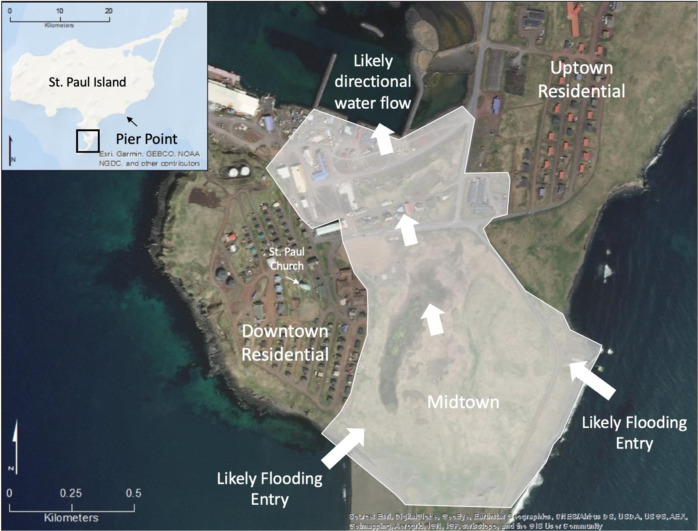
Pier Point, Midtown, Church. White shaded area indicated the portion of the town that is vulnerable to erosion and flooding due to low elevation. White arrows indicate the likely directional flow of water across town during an intense flooding event

In addition to the six main areas identified by interviewees, there were several areas of concern that few residents identified where erosion and changes on the island have been observed with some frequency. These locations included the community Russian Orthodox church located centrally in town; Pier Point, a landmark on the island where subsistence harvesting occurs; and Midtown, referring to the low-lying land that separates the two residential areas of town (Fig. 6). Each of these locations were only mentioned by one or two interviewees each, although described in detail by each individual, thus they were considered to be secondary areas vulnerable to erosion and are not further discussed in this study. However, it is important to note that these secondary areas were related to other higher priority areas because of their shared connections to either culture and tradition and/or subsistence.

Beyond locations, four broad themes emerged from our coding analysis: erosion, including observed changes and future potential changes, the timing of erosion events being observed, and erosion as a new or emerging issue; access to locations of subsistence and cultural importance; mitigation efforts that had recently been completed or were perceived as needed; and concern or worry regarding the impacts of erosion (in conjunction with other natural and anthropogenic environmental changes). Erosion was most often cited as “caused by” climate changes including sea level change or rise; increased storm surges, high winds and wave activity; a lack of ice and snow; and warming air and ocean temperatures. Of the 103 times that “erosion” and erosion-related terms were mentioned by interviewees, the terms co-occurred with the terms listed above describing climatic changes 41 times. Erosion was discussed as causing the most damage through erosion of sand dunes, collapsing of cliffs or bluffs (such as the backside of the cemetery), and accretion of sands in low-lying areas and roadways. “Worry” or “concern” due to erosion impacts, in regard to both observed changes and anticipated future changes, was a major theme and was associated with location-specific subsistence and cultural impacts and generally related to negative social impacts. “Subsistence hunting” was central to many of our conversations and linked strongly to the issue of access to important traditional foods (marine mammals, seabirds, and waterfowl). Erosion was cited as a priority threat to food security that must be mitigated, even though individuals felt local entities (ECO, the City, and TDX) would not address it until they absolutely had to (i.e., loss of access versus threatened loss of access).

### Focal Study Questions

#### What are the community’s perceptions of erosion on St. Paul in the context of the island’s other environmental concerns?

This question provided insight on how much or little residents prioritized erosion in relation to other environmental concerns. Previous studies have shown that individual perceptions vary widely depending on the number of climate stressors, degree of urgency, perceived risk, and attribution of climate versus social or economic stressors (Dinero 2013; Herman-Mercer et al. 2016; Ambrosio-Albala and Mar Delgado-Serrano 2018). Respondents generally noted a definitive timeline when they began noticing erosion occurring on St. Paul and an increase in changes to the environment in recent years. These changes were related to other climate changes being perceived by residents, which is consistent with similar studies (Ambrosio-Albala and Mar Delgado-Serrano 2018; Marin and Berkes 2013). A majority of interviewees viewed erosion as a concern with potentially devastating consequences, as noted by high occurrences of the word “erosion” and erosion-related terms compared to other community concerns (i.e., decline in winter snow cover, declines in wildlife populations, shifting social behaviors, stochastic events). Erosion, however, was not viewed as being so urgent that it required immediate attention as compared to other community concerns. One of the initial questions asked in all interviews was, “Have you seen any changes on the island over your lifetime?” (Table 1). The most common responses regarding observed changes referred to the island’s declining wildlife, general societal changes within town, and weather, including changes from historical trends and increasingly unusual weather activity in recent years. According to one resident who has lived on St. Paul since the 1960s, “The seals are declining, the birds are declining,…” and they recalled how, “…we used to go a little ways out there [offshore] and drop our line to catch halibut, sometimes now we got to go 12 miles around the island to catch halibut” (interview, 24 July 2019). Another resident described how St. Paul used to have various recreation spaces for youth that are now no longer available and continued to describe how “…grocery prices have gone way up since we were younger, too” (interview, 26 July 2019). Residents who were primarily concerned with unusual weather described how the summer is “…hotter by 10 °F. We never get to the 60s, and we did this summer. Usually it’s in the 40 and 50s, but not in the 60s” (interview, 17 July 2019). There was considerable overlap in interviews where individuals talked about erosion along with these other prominent concerns, with shifts in human behavior and changes in weather commonly viewed as correlated with erosion by individuals.

Compared to these other key issues, erosion was identified as a top priority or urgent concern for four residents. One resident acknowledged a lack of prioritization and said, “With all that’s happening with the environment today, erosion should be at the top of the list. Are we doing everything we can today to monitor it? Probably not. But we’re not experienced with a bunch of life-threatening things here. A lot of our shores are volcanic and sand and we’re not seeing devastation in these areas” (interview, 15 July 2019). While collectively the community understands how potentially life-threatening erosion is to their livelihoods, many residents lack an immediate compulsion to act. This is reflected as a “lack of action” around the mitigation theme that emerged from our coding analysis, where only a few instances describing St. Paul local entities’ general lack of action to address or mitigate erosion included knowledge that there are some mitigation measures that are occurring currently (i.e., road repairs at Benson Beach, Big Lake, and Zapadni haulout).

Understandably, the community lacked a sense of urgency considering the timescale that erosion on St. Paul functions in relation to an individual’s lifetime or generational timescale. Erosion can operate at multiple and unpredictable scales, taking decades or longer to cause more than a few millimeters of loss, or causing devastation fully in the span of days to years (Nearing et al. 1999; DiBiase and Whipple 2011). While residents may acknowledge that erosion is an issue on the island, in their daily lives, the environment may look the same and erosion go undetected until a sudden large change occurs or until they reflect on what an area looked like years prior. One resident exemplified this sentiment when talking about the cemetery, “We moved the cross in [from the bluff] 20 years ago, and now it’s [bluff edge] reaching the cross again. Last year [2018] I moved it inland another 20 yards” (interview, 1 August 2019).

Access to subsistence resources was another key theme related to erosion impacts. The St. Paul community is deeply rooted in the Pribilof Islands ecosystem and draws their cultural identity from the island’s wildlife, chiefly northern fur seals, so it is understandable for the community to be urgently concerned about observed wildlife declines of important subsistence and commercial resources (e.g., Pacific halibut *Hippoglossus stenolepis*, snow crab *Chionoecetes opilio*), rather than erosion in and of itself. Northern fur seal abundance on the Pribilof Islands has declined dramatically since the 1950s, with an annual rate of decline of 4% since 1998 (based on pup production, a primary metric of abundance; Towell et al. 2016). As Pribilovian Unangax̂ self-identify as “People of the Seal”, and consider themselves responsible for protecting and being stewards of the Pribilof Islands fur seal population, it is not surprising that tribal members are much more aware of changes associated with the continued decline of the fur seal population. Additionally, recent unusual mortality events of various seabird species, including tufted puffins (*Fratercula cirrhata*), short tailed shearwaters (*Puffinus tenuirostris*), and northern fulmars (*Fulmarus glacialis*), are creating a stark visual for residents who are noticing the sudden abundance of bird carcasses. This is likely to induce a visceral reaction and could expedite wildlife declines as being perceived as a priority issue and a growing concern for residents.

In addition to the overall perceived lower priority concern for erosion, the community may not appear as concerned about erosion as compared to other environmental issues because of a sense that individuals can do little to prevent erosion. Some of the most commonly identified environmental concerns have concrete measures that can be pursued and implemented, giving residents a sense that they can affect positive changes. For example, in response to observed wildlife population declines, residents can advocate for conservation and management actions such as issuing fewer hunting permits, adjusting fishing quotas, and creating protected marine areas. These actions provide a level of comfort to individual community members because they are rooted in a sense of tangible accomplishments. In contrast, residents may know that there are specific measures being undertaken by various local entities in regard to erosion, but it presents more abstract effects as a result climate change. There is no clear single solution to address the issue, which can make erosion adaptation seem daunting; additionally, monitoring activities can reinforce the feeling that minimal mitigation or adaptation measures have been taken (ECO, unpub. data). As one permanent resident who has lived on St. Paul for over 20 years described, “Nature’s going to do what it’s going to do… if you try to fight it, it never goes well” (interview, 23 July 2019). Elders shared the same feeling of being a passive observer; one Elder half-joked, “What are you going to do? Protest the wind? “Stop [wind]!” Ha!” (interview, 22 July 2019).

Residents’ observations of social changes occurring on the island were also perceived as a higher priority concern, because St. Paul has greatly changed over the past 50 years. Elders noticed that younger generations seem to be more financially stable compared to previous generations. When discussing the importance of subsistence hunting, one resident described how “A lot of times it was kinda hard to get money in, so if you wanted to get something, you had to go out and work for it. Nowadays some parents are a lot wealthier than what we grew up with, so they are able to get stuff [from the store]” (interview, 24 July 2019). Some residents also expressed concern over how young adults are emigrating both temporarily and permanently. Many youths on St. Paul leave upon entering high school to attend one of two in-state boarding schools in pursuit of a perceived more rigorous education and to gain exposure to new experiences beyond the island. The concern of continued or increasing emigration over time has been prominent in the community since the 1960s (Foote et al. 1968). With these economic and demographic changes shifting on St. Paul, there has been a generational difference among community members where youth inherently have different experiences and perceptions of the island compared to Elders. The shift in the way of life has caused worry among residents about how the community will be able to preserve Unangan culture and traditions in the future, making societal and cultural observations understandably a higher priority than long-term erosion concerns.

Another reason that residents seem to lack a sense of urgency on erosion can be attributed to their awareness of more immediate concerns. Most residents noted the increase in unusual weather and discussed warmer temperatures, less snow, and increased storm surges occurring in recent years. Each of these observations center on more immediate life-threatening concerns rather than long-term issues like coastal erosion. An issue that several residents raised in relation to weather changes is the island’s source of freshwater. The groundwater on St. Paul is partially replenished by snow and residents commented on the alarming lack of snow in the past several years. As one resident said, “For me, and my opinion, environmentally our main concern is water- freshwater. Who knows where our water source is gonna be in the future”, continuing to state, “I mean in the last 6 years we haven’t really had any winters so we aren’t really accumulating all that snow melt off [runoff]. And we are not getting enough rain to replenish [the water table]” (interview, 15 July 2019). A loss of the island’s water supply would create an immediate crisis for residents. Thus, it is not surprising that this issue creates a more immediate concern for residents as erosion continues to be a chronic problem that has yet to seriously affect the community.

Regardless of the relative priority that the community places on erosion, residents are still very aware of how the pace of erosion has been accelerating in recent years. One resident noted that, “In the last 5 years we’ve seen a lot of erosion. Some of the beaches on the roads are caving in, mostly in just the last few years it changed a lot” (interview, 17 July 2019). Another resident hypothesized that, “Erosion is increasing. It might move a lot quicker now that we’re receiving a lot more storms in the fall, and the storms are hitting a lot sooner [in the season] and stronger” (interview, 24 July 2019). With this mounting concern, the community would like to see proactive measures being taken. One interviewee described how at a previous tribal council meeting, a council member brought up the idea of relocating the community cemetery, and one of the Elders exclaimed, “You can’t move people! You can’t do this!” (interview, 23 July 2019). This passionate debate is fueled by sense of history and connection to one’s ancestors, and simply discussing various mitigation options can be uncomfortable and lead to divisions within the community. Some residents would like to see proactive measures taken now to move the cemetery to a less vulnerable area, but relocation would require approval from landowners, likely TDX who acquired a majority of the land on St. Paul through the Alaska Native Claims Settlement Act. Relocation would also require the pooling of capital resources and additional capacity from multiple local and external entities in addition to receiving approval from family and tribal members, which is currently a heated debate.

Based on our interviews, erosion is one of many important issues that the community faces. It has been a source of worry and stress for residents because of its slow progression over time and lack of tangible action. While other issues may take priority in the immediate future, erosion continues to wear on the minds of residents and presents a seemingly never-ending problem with complicated solutions. This is idea is also known as solastalgia, or the distress caused by environmental change to a people who have had a long-established connection to their home environment (Albrecht et al. 2007).

#### Does the community’s understanding of erosion mitigation reflect the actual work done by local entities?

This second question sought to consider how the community understands erosion mitigation and adaptation and how the local entities responsible for it on St. Paul are addressing the issue. Those interviewed seemed to feel a sense of frustration with the three main local entities involved in erosion mitigation—ECO, the City of St. Paul, and TDX—as interviewees described their frustration in 41 instances. One interviewee in particular who has worked for the Tribal Government for nearly two decades described how the entities are, “…going to wait until it’s down to the bare minimum before they do something [because] they all got their own ways of working. It’s not like the old days where you can have a pow wow inside an office and go around to shake a hand and get things done” (interview, 25 July 2019). When discussing with an Elder about how costly it would be to construct a possible alternative road around the vulnerable area near Zapadni haulout, as has been previously proposed as a mitigation measure, the individual threw their arms in the air and shouted, “I want [the road] to go all the way around. I don’t care [how much it costs]! People love their seals and will do anything for them” (interview, 23 July 2019). Many residents expressed a sentiment of need for coordination and collaboration among the three local entities. Often, residents stated that “they” should act on the erosion issue, which always implied a combination of ECO, TDX, and the City, but never a specific individual entity. Petheram et al. (2014) found that similar expectations of government entities were held by residents in South Goulburn Island, Australia where a feeling that government agencies should serve a primary role in adaptation planning assistance dominated.

However, after interviewing staff from each organization, it became apparent that each entity’s actions are relatively disconnected and independent of one another. For example, when discussing who is responsible for maintaining the roads that provide access to subsistence hunting grounds and recreation areas outside of the immediate town vicinity, TDX staff indicated responsibility lies with the City, while the City indicated responsibility is held by TDX (interviews, 23 July 2019; 1 August 2019). Lacking a common understanding of their respective roles and responsibilities led to gaps in management on the island which have been directly observed by residents.

The discrepancy in residents’ understanding of what roles and responsibilities each local entity has can also at least partially be attributed to a lack of transparency and access to the monitoring information. In “The City of St. Paul’s Strategic Plan for 2017–2020”, one weakness noted was a greater need for communication with residents and a lack of understanding as to what the City does (Agnew::Beck Consulting 2017). Additionally, neither the City’s nor the Tribal Government’s websites contain any information on their respective erosion mitigation efforts, like refilling areas of loss and creating alternative access routes to access locations of importance. Beyond improving communication with residents, communication among the entities monitoring and managing erosion issues can be much improved. After learning about “St. Paul’s 2016 Hazard Mitigation Plan” through an interview, several ECO staff members noted they were previously unaware of the document’s existence (ECO, pers. comm.; City of St. Paul Hazard Mitigation Planning Team 2016).

The lack of understanding among local entities can also be ascribed to the complexity of coordinating erosion monitoring and mitigation across local managing entities, each with their own priorities, roles and responsibilities, and budget constraints. Since each organization is responsible for providing various services on the island, there is no single coordinating body to organize and streamline erosion monitoring and mitigation on the island. The lack of centralization leads to confusion that can stall monitoring, mitigation, and adaptation planning efforts.

A final source of frustration could be due to the cyclical cycle of concern and lack of action. Several residents described how erosion has been a topic of concern for decades, yet many of the solutions implemented thus far have been short term and temporary. As one interviewee stated, “…we’ve always talked about it, and nothing’s come about it…everybody’s out for the bottom line. ‘How much can I charge you?’” (interview, 25 July 2019). Another interviewee supported that sentiment by questioning, “Why couldn’t they just do it for respectfulness? Just do it for everybody in the community instead of doing it for the money?” (interview, 26 July 2019). By going through the cycle of discussing erosion issues among the relevant entities, yet not taking preventative measures, a sense of distrust grew between residents and the local organizations. Both residents and local agencies are aware of the erosion occurring on St. Paul, thus leaving residents questioning the agencies’ lack of concrete action.

While the community lacks a full and complete understanding of the work that the three entities are doing to mitigate erosion, each organization also lacks a full understanding of what the others are working on. The residents’ sense of skepticism in what “they” are working on to address the issue seems to stem from a more general sense of distrust not specific to coastal erosion. This sense of primary responsibility being inherent to government and other institutional bodies, paired with mistrust from the community, has been documented in other Indigenous communities facing climate impacts (Petheram et al. 2014; Ali et al. 2019). Additionally, in the case of St. Paul, a gap in understanding each organization’s capabilities, limitations, resources, and roles was also documented, thus demonstrating how there is room for improved coordination among the three entities.

#### How does erosion relate to and impact local Unangan cultural traditions and heritage?

The final research question allowed us to expand and recognize how erosion directly impacts the community’s long cultural traditions, especially considering how culture, identity, community cohesion, and sense of place are vulnerable to climate changes (Blennow et al. 2019). The St. Paul community’s devotion to maintaining ties to its culture, traditions, and history is directly related to a strong sense of place. Sense of place refers to more than the physical location of an area; it is the emotional connection one has to a place and the values, symbols, and cultural meanings associated with the area. Developing a sense of place is a personal experience unique to each individual (McCunn and Gifford 2018) but these personal experiences are tied to an Indigenous context where “place” is also connected to one’s identity, health, and culture (Walters et al. 2011; Blennow et al. 2019). Seeing places change over time due to ecological or physical processes that are in no one’s control can be detrimental to an individual and can be equivocated to a loss of spirit or identity (Snyder et al. 2003; Walters et al. 2011). As disturbances affect the physical characteristics of a place and what it means to an individual, this leads to emotional or psychological distress (Masterson et al. 2017). Therefore, potential worsening erosion on St. Paul can directly alter one’s sense of place and cause harmful distress since it can weaken one’s bond and connectedness to their environment.

All six of the identified priority locations of erosion concern represent areas that, if severely eroded, could result in potential losses related to Unangan cultural traditions, heritage, and sense of place. Specifically, residents were concerned about the island’s cemetery as a place of reverence and communion. Cemeteries serve as symbols of the distant past of a society and are indispensable for the remembrance of social events (Basso 1996). Losing this critical culturally significant site would result in the loss of almost all burial sites for permanent residents since the island’s settlement during Russian occupation (late 1700s–1800s). Additionally, the cemetery is still used today, and many residents feel a deep sense of cultural connection with the area since it continues to serve a critical role in the mourning and grieving process. Some residents’ fears of the cemetery eroding away have been present for decades. As one interviewee recounted, “I told my grandma that she couldn’t be buried at the top of the cemetery because I didn’t want to fish her bones out of the water. Such a random thing for such a young child to say, but I said that to her all the time” (interview, 26 July 2019). This sense of worry has also affected the younger generation; as one youth described, “I always hear older people talking and telling me that someday the cemetery is going to fall, and bodies are just going to come out. I believe it” (interview, 26 July 2019). Fear of the loss of the cemetery was further supported by one resident who said that the cemetery is “…our history… Our families are buried there, so when you’re up here and you have to dig to bury loved ones and you see the backside of it [the cemetery] going [eroding], it makes you stop and think” (interview, 25 July 2019). Losing the cemetery would greatly impact the community’s cultural uniqueness and sense of place, especially considering the small population of St. Paul (~450 residents). Regardless, relocating the cemetery may have to be considered in the future if changing weather conditions continue to erode the bluff where the cemetery is located.

In addition to the cultural importance of the erosion areas, subsistence hunting, and harvesting is also key components of Unangan culture that help the community obtain sustenance and maintain traditional ways of life, thus making access and ability to hunt and harvest animals paramount for the community. Of the six areas of erosion concern that residents identified during this study, five are directly tied to subsistence hunting and harvesting and their importance was mentioned by residents 87 times. Sea Lion Neck, Benson Beach, the road adjacent to Big Lake, Zapadni haulout, and Reef Rookery all provide access to hunting grounds for subsistence species that the community relies on. One resident who grew up hunting said that erosion will “…cut down on the way of life out here for a lot of people. A lot of food, a lot of natural resources. Birds, seals, fishing…” (interview, 25 July 2019). Referring to Reef Rookery, an Elder waved their hand out toward the horizon as they said that the area is used “mainly for subsistence hunting, we get everything there. Sea lion, seal, pups, kittiwakes, winter ducks, you name it” (interview, 23 July 2019). In the case of northern fur seals, a species that defines the culture and community on St. Paul and is one of the most important subsistence harvested animal on St. Paul, one resident remembered how the place where they used to hunt and harvest has changed over time. “That used to be our killing ground right there, where Zapadni Beach is. We used to hunt right there, but now with the grass and how far the wave action is, it took the whole area out” (interview, 1 August 2019). The areas identified here represent those locations with the most obvious observed changes due to erosion that may affect subsistence resources, and thus should comprise the focus for near term adaptation and mitigation efforts in the community.

When asked who taught them how to hunt, all interviewees said that they learned from their parents, aunts, uncles, grandparents, or other family members. Passing on hunting values and skills is a critical part of producing future generations of responsible and knowledgeable subsistence hunters on St. Paul. Without subsistence opportunities, the ability to transfer these skills is vulnerable to loss. One resident described how they used to hunt as a child and passed the skill on to their nephew, recalling, “I grew up going out there hunting Steller sea lions and ducks. Without that access to that [Northeast] point, it’s an area we won’t be able to hunt” (interview, 24 July 2019). Another resident reminisced about their fond memories of hunting and described how “Sometimes the whole family goes out and goes out to northeast [to hunt]. So, I think that’s pretty good, [the kids] getting a view of how to hunt so when they’re younger if they pick it up, they probably have a better chance of continuing on when they get older” (interview, 24 July 2019).

Beyond maintaining and passing on important cultural practices to younger generations, access to subsistence resources is critical for residents to obtain nutritious foods throughout the year. Grocery store foods have a long and complicated journey to St. Paul, and like many rural Alaskan villages, prices for goods are frequently over twice the retail cost of foods in larger urban centers. Produce, in particular, experiences high spoilage rates (i.e., > 65%) during the 7–14 day wait on cargo planes in Anchorage, Alaska, before reaching St. Paul (ECO, unpub. data). In contrast, subsistence foods available on St. Paul, including marine mammals, fish, roots and berries, are high in key nutrients and have sustained Unangax̂ for hundreds of years since permanent settlement of the island.

**Fig. 7:**
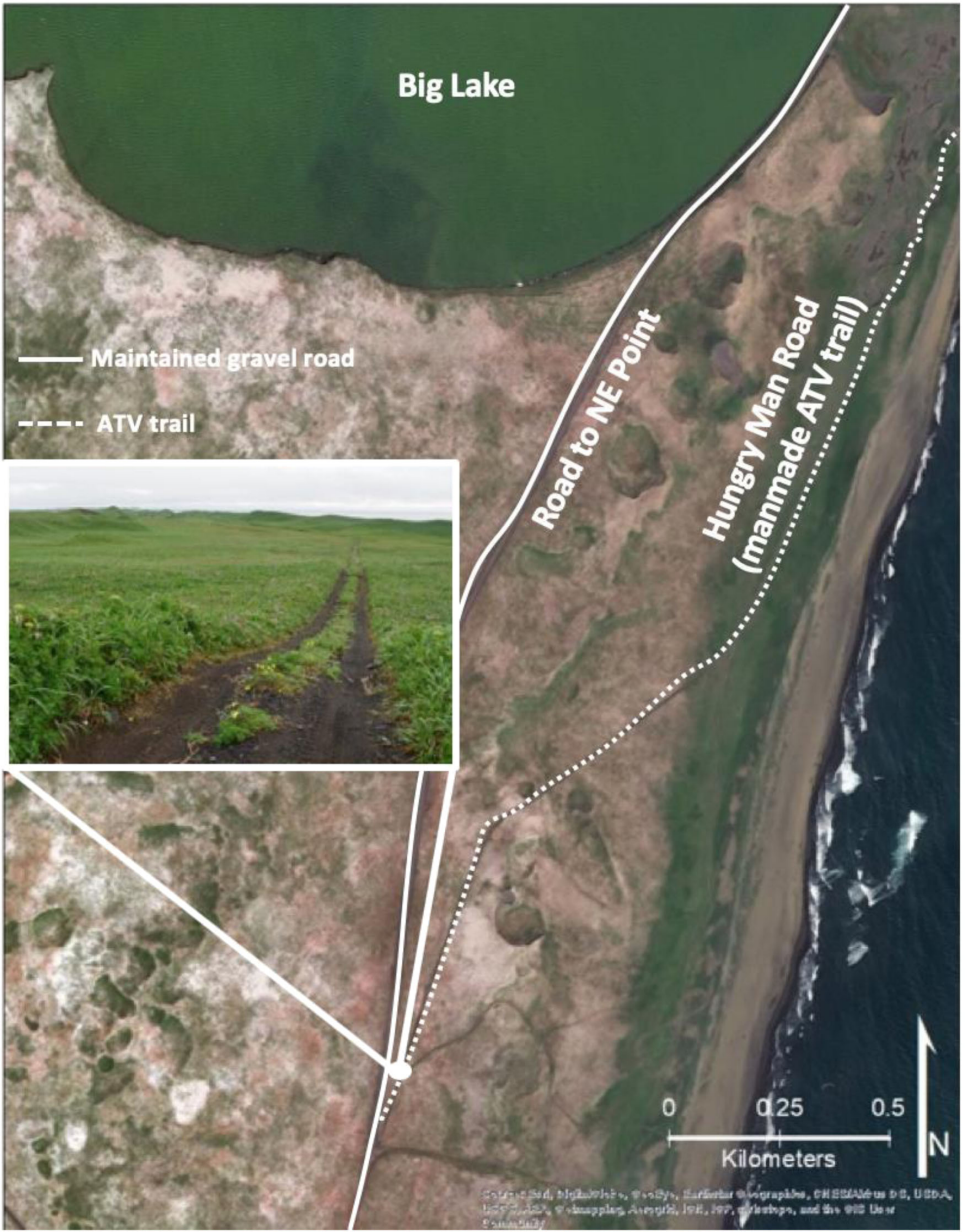
Hungry Man Road at the northeast region of St. Paul Island. The inset image shows the trail that was created by ATVs over years of use when access to the road was impossible due to accumulated snow and ice. Deep rutting from ATVs and four wheel drive vehicles are vulnerable to erosion

Despite how erosion may potentially weaken the community’s ability to access key resources and the ability to pass on traditions to future generations, many residents have exemplified resilience in the face of this threat. We asked residents, “What would happen if you couldn’t access these areas to hunt or harvest?” and one of the Elders who was quiet throughout the entire interview earnestly stated, “That wouldn’t happen!” (interview, 23 July 2019). Another Elder commented that, “There will always be an area where we can slaughter them [subsistence harvested northern fur seals]. If it did happen, I would shoot them myself and hunt them like we hunt sea lion” (interview, 23 July 2019). Another illustration of the community’s determination to continue accessing their traditional food sources can be shown through the creation of Hungry Man Road (Fig. 7), an off-road vehicle trail that was created by subsistence hunters during times when Benson Road, the only maintained road to the northeastern region of St. Paul, was covered in snow drift or ice. As one resident described, “That’s why Hungry Man Road was created. If you’re hungry and wanted sea lion, you have to go this way” (interview, 15 July 2019). Hungry Man Road, however, is not always safe, as observed by a community member, “There’s a treacherous area. There’s a big hole here, sand washed out, and then there’s another one [hole], so you have to drive at an angle like this [motioned sideways with their arm at an angle]!” (interview, 1 August 2019). Even today, the subsistence hunters we interviewed were steadfast with their confidence that if there is a will, there is a way. This vivid determination to continue accessing the same resources that their ancestors utilized exemplifies the community’s mentality in combating the impacts of climate change including erosion impacts. Regardless of what will happen, some residents hold a resolute confidence that they will somehow be able to continue their traditional ways of life. Indeed, it is this characteristic of resolution to adapt and thrive that is most notable in Alaska Native cultures.

In addition to observations of how erosion affects their connection to the cemetery and subsistence hunting areas, St. Paul residents also commented on how erosion has affected their emotional connections to the island overall. One resident posited, “It’s basically just kind of like watching a playground get torn down. I’ve played all over the place on St. Paul everywhere, on cliffs and things like that, dangerous things, but you take a walk around town and look at them as an adult and you can notice everything you noticed as a kid- gone” (interview, 26 July 2019). Similarly, many residents shared sentimental memories from some of the places that are most threatened by erosion. For example, Zapadni haulout holds a special place in one of the young interviewee’s consciousness. After joyfully telling a colorful story of how the individual’s family went on a trip there, the youth commented that they would, “feel very sad [if Zapadni haulout eroded away] because that’s a place where you can look at seals and feel very safe” (interview, 24 July 2019). Indeed, many residents hold sentimental memories and feelings of security tied to a specific set of locations on St. Paul. On a similar note, one of the adult interviewees described how, “I used to be really excited to go out [to Northeast], but now I absolutely refuse to look over by the lake because I don’t like to see how close we are towards the edge”, and continued to say, “It’s sad to see things changing, I mean things are always going change but you don’t like to see things changing for the worse” (interview, 26 July 2019). With the current and worsening visually obvious erosion occurring on St. Paul, many residents developed altered views of the future of St. Paul; one Elder solemnly stated, “I think St. Paul will no longer be sand” (interview, 22 July 2019), highlighting how quickly beaches are eroding away. These thoughts of how the island has changed over time, or may change in the future, demonstrate how the effects of erosion have created a changed sense of place for at least some residents. For some residents, the emotional connection that they once had with the island has manifested into a sense of worry and fear of how St. Paul will look in the future. Recognizing that the original settlement of St. Paul is rooted in slavery and colonialism, there is an already present intergenerational trauma that weighs on and affects residents. Despite being forcefully relocated from the Aleutian Chain to the Pribilof Islands, Unangan developed a strong enduring cultural identity specific to St. Paul. Erosion adds to these other stressors on the community and has produced as yet unknown effects on residents’ sense of place.

St. Paul residents have always exemplified resilience in adapting and creating solutions to erosion, but we must recognize and call attention to the extreme emotional toll of losing one’s sense of place. Residents have very strong attachments to St. Paul, attributed to deep enduring ancestral and cultural connections to the land and sea; reliance on subsistence activities for biophysical, emotional, and cultural health and well-being; and a multifaceted reverence and understanding of the natural environment. The threat of losing critical regions on St. Paul due to new or worsening erosion would dramatically alter the community since these changes could undercut the emotional and psychological basis of their sense of place and home. Even though residents may still be located on St. Paul, the slow progression of change may necessitate adjustment and adaptation to a different location than where residents have previously thrived.

## Conclusions and Next Steps

By conducting interviews through semi-guided conversations, residents detailed specific observations, and concerns that they had about erosion on St. Paul, expressed their thoughts on how local entities were managing and mitigating erosion, and explained how erosion has affected local cultural traditions and heritage. The information from this study will aid the community of St. Paul in guiding next steps to proactively address local erosion impacts and can inform other Indigenous communities in similar situations to shape future erosion monitoring, mitigation, and adaptation planning activities. In addition, while other studies have documented Indigenous perceptions of climate impacts, this study is unique in that it also analyzes residents’ perceptions of the local actions being taken, or not taken, to address the issue, and the perceived roles and responsibilities of local governing entities.

Erosion was generally recognized by interviewees as a threat to the community, with varying perceptions of its urgency and need for response. Residents’ attitudes were placed along a spectrum of feeling that the community is resilient and will always rely on their ingenuity to find solutions, to a fatalistic feeling where there is little that can be done to combat environmental changes and belief that actions taken will not result in tangible outcomes. These viewpoints were also placed in context of competing with other priorities that were perceived to be equally or more urgent, such as changes in important subsistence species. From our study, residents also expressed frustration with the local entities responsible for addressing erosion concerns due to the perceived lack of action and coordination, paired with a misunderstanding of the roles, capacity, resources, and limitations held by each entity. Throughout the study, residents continually expressed how erosion threatens more than just land, as it potentially detriments their ability to maintain their culture, heritage, traditions, and sense of place.

From our conversations with St. Paul residents and drawing from the literature, we identified several actions that can serve as initial steps in climate adaptation and mitigation planning. The six locations on the island that were identified as high risk are a logical starting point for implementing locally coordinated mitigation and adaptation efforts. Local entities can also draw from the community perceptions documented in this study to better understand how to target and improve outreach to residents, enact appropriate and community-driven mitigation measures, and better collaborate with one another in addition to state and federal agencies. There is certainly room for improved collaboration on erosion issues, both among intra-island entities and across the state, to combine resources and streamline information sharing, rather than continuing to work independent of one another. In the immediate future, we recommend developing an advisory committee specifically to address erosion concerns and to improve communication and collaboration among key local entities. A shared committee will enhance improvements through shared capital, capacity, and information on erosion issues in order to facilitate improved and efficient mitigation efforts. Second, we recommend the locally created advisory committee identify the highest priority erosion locations and establish appropriate mitigation measures, especially for locations critical for maintaining access to subsistence hunting and harvesting areas.

We also suggest that the community finalize a working climate adaptation plan that can be implemented as soon as possible and reevaluated on an ongoing basis. We recommend the plan define the community’s expectations and goals to achieve in the short term (i.e., next 3–5 years), establish mitigation plans for locations of highest priority, and provide concrete roles for entities and steps to achieving these adaptation and mitigation goals. The plan should account for the best practices we found in our literature review, such as valuing Indigenous and local knowledge equally to western science (Huntington and Watson 2012; Tribal Adaptation Menu Team 2019); making the plan adaptable to address current stressors and systems that present barriers to community well-being (Ford 2012; Wilson 2014; Loring et al. 2016; Marino 2018; Huntington et al. 2019; Tribal Adaptation Menu Team 2019); building in a process for continual community input and feedback, particularly from Elders and subsistence hunters (Klenk and Meehan 2015; Johnson et al. 2016; Tribal Adaptation Menu Team 2019); and designing it to be as specific to the community’s culture and history as possible (Tribal Adaptation Menu Team 2019).

These short- and long-term actions can apply to other communities facing emergent climate-related concerns. We recognize that many of these steps require considerable time, resources, and capital that is not always flexible or available. Given this, we suggest that communities seek out existing institutional partners to connect to new and additional resources more immediately. Though a 2016 publication pointed out a lack of tools for bottom-up, community-driven adaptation planning for Indigenous communities (Carmichael et al. 2017), in recent years, resources have been in development and are becoming more readily available, but a lack of awareness of what tools exist and how to access them likely present a current challenge. For example, Indigenous communities in Alaska and throughout the USA can obtain information on how to begin erosion monitoring from the State of Alaska erosion monitoring program through the DGGS; the Alaska Native Tribal Health Consortium can help communities identify potential funding opportunities and build community capacity; the US Bureau of Indian American Affairs Tribal Resilience Program has competitive funding opportunities; the Institute for Tribal Environmental Professionals has an Adaptation Planning Toolkit and training opportunities; and the US Climate Resilience Toolkit has over 200 digital tools related to climate adaption and mitigation planning. For communities outside of the USA, other Arctic resources and supporting institutions include: the Arctic Council and Inuit Circumpolar Council, which has developed assessments, factsheets, and working groups; the Arctic Institute of Community-Based Research that has supported numerous studies and community-based plans in Canada; the Indigenous Leadership Institute who helped to expand the Indigenous Guardians program across Canada; the Assembly of First Nations, which advocates on behalf of Canadian First Nations on climate change policy, facilitates dialog and campaigns, and liaisons with governments; and the Snowchange Cooperative based in Finland which created a network of communities across the Arctic focused on climate adaptation and sustaining Indigenous ways of life. Partners like these listed above can assist communities by identifying and connecting communities to a variety of resources, and they can be incredibly useful in informing communities on how to start taking actions to move forward.

While many aspects of this study can be adapted to other Arctic Indigenous communities, one quality that cannot be transferred is an understanding of how a community values and connects the physical environment to their cultural traditions and heritage. There is no equivalent to listening directly to residents as they detail in their own manner their place-based connections to their lands and waters, resources, and traditions. As highlighted in the climate adaptation literature, Indigenous and local knowledge—such as what was documented in this study—is fundamental to the planning process in order to ground planning efforts and mitigation strategies in place-based connections, cultural and historical significance, and community dynamics (Cochran et al. 2013; Ignatowski and Rosales 2013; Manrique et al. 2018; Richards et al. 2019). This study showcases how community involvement can be used as a successful tool to both engage community members in the early stages of the adaptation planning process and incorporate community-sourced information to inform next steps and strategies, a key tenant in Indigenous-based planning (Klenk and Meehan 2015; Johnson et al. 2016; Tribal Adaptation Menu Team 2019).

Understanding how the community perceives and understands environmental changes is key to implementing successful adaption measures since they lead to numerous benefits such as helping inform priorities for future actions, facilitating trust between residents and local decision-makers, and empowering community members to be involved in creating solutions and possibly help negate feelings of frustration or despair. Perceptions of risk and urgency of specific climate impacts are informed by cultural dimensions unique to each community, and as such can inform adaptation planning in terms of identifying interventions and actions, determining the timescale for action, and how best to allocate funds and resources. More specifically, when addressing emergent climate impacts—as opposed to acute or long-standing impacts—developing shared knowledge between organizations, institutions, and community members will be especially useful since it can enable the community as a whole to be proactive, if so desired and possible. This in turn can yield more time to develop creative and thoughtful solutions that can be implemented before more disruptive and costly policies (e.g., relocation) become necessary.

## Data Availability

Metadata and relevant files are available by request to the Ecosystem Conservation Office at lmdivine@aleut.com. Raw audio files of interviews and focus groups are not available as they are confidential.

## References

[CR1] Agnew::Beck Consulting (2017) City of Saint Paul Strategic Plan: 2017-2020. http://www.stpaulak.com/download_file/view/135/328. Accessed 1 Aug 2019

[CR2] Alaska Native Tribal Health Consortium (ANTHC) (2018) Newtok Relocation Quarterly Update. https://s3-us-west-2.amazonaws.com/ktoo/2018/12/Newtok-Relocation-Quarterly-Update_2018-Oct_FINAL.pdf Accessed 4 July 2020

[CR3] Albrecht G, Sartore GM, Connor L, Higginbotham N, Freeman S, Kelly B, Stain H (2007). Solastalgia: the distress caused by environmental change. Australas Psychiatry.

[CR4] Ali MF, Ashfaq M, Hassan S, Ullah R (2019). Assessing indigenous knowledge through farmers’ perception and adaptation to climate change in Pakistan. Pol J Environ Stud.

[CR5] Ambrosio-Albala DP, Mar Delgado-Serrano DM (2018). Understanding climate change perception in community-based management contexts: perspectives of two indigenous communities. Weather Clim Soc.

[CR6] Armitage D, Berkes F, Doubleday N (2007). Adaptive co-management: collaboration, learning and multi-level governance.

[CR7] Basso KH (1996). Wisdom sits in places: landscape and language among the West Apache.

[CR8] Berkes F (2012) Sacred ecology. Routledge, New York, USA. 10.4324/9780203123843

[CR9] Black L (1983). Some problems in the interpretation of Aleut Prehistory. Arct Anthropol.

[CR10] Blair B, Lovecraf AL, Kofinas GP (2014). Meeting institutional criteria for social resilience: a nested risk system model. Ecol Soc.

[CR11] Blennow K, Persson E, Persson J (2019). Are values related to culture, identity, community cohesion and sense of place the values most vulnerable to climate change?. PloS ONE.

[CR12] Boillat S, Berkes F (2013). Perception and interpretation of climate change among Quechua farmers of Bolivia: indigenous knowledge as a resource for adaptive capacity. Ecol Soc.

[CR13] Bronen R, Chapin FS (2013). Adaptive governance and institutional strategies for climate-induced community relocations in Alaska. Proc Natl Acad Sci USA.

[CR14] Brubaker M, Berner J, Bell J, Warren J (2011) Climate change in Kivalina, Alaska strategies for community health. Alaska Native Tribal Health Consortium. http://www.cidrap.umn.edu/sites/default/files/public/php/26952/Climate%20Change%20HIA%20Report_Kivalina.pdf. Accessed 24 July 2019

[CR15] Buzard RM, Overbeck JR, Maio CV (2019) Community-based methods for monitoring coastal erosion: Alaska Division of Geological & Geophysical Surveys (DGGS) Information Circular. 10.14509/30182

[CR16] Callaghan TV, Kulikova O, Rakhmanova L (2020). Improving dialogue among researchers, local and indigenous peoples and decision-makers to address issues of climate change in the North. Ambio.

[CR17] Carmichael B, Wilson G, Namarnyilk I, Nadji S, Cahill J, Bird D (2017). Testing the scoping phase of a bottom-up planning guide designed to support Australian Indigenous rangers manage the impacts of climate change on cultural heritage sites. Local Environ.

[CR18] Carothers C, Brown C, Moerlein KJ, López JA, Andersen DB, Retherford B (2014). Measuring perceptions of climate change in northern Alaska: pairing ethnography with cultural consensus analysis. Ecol Soc.

[CR19] Church JA, Clark PU, Cazenave A, Gregory JM, Jevrejeva S, Levermann A, Merrifield MA, et al. (2013) Sea level change. Climate Change 2013: the physical science basis. Working Group I contribution to the 5th Assessment report of IPCC. Cambridge University Press, Cambridge, UK

[CR20] City of St. Paul (2019) Government. http://www.stpaulak.com/government. Accessed 3 Sep 2019

[CR21] City of St. Paul Hazard Mitigation Planning Team (2016) City of St. Paul, Alaska Hazard Mitigation Plan. City of St. Paul. https://ready.alaska.gov/Plans/documents/Saint%20Paul%20Draft%20Plan%202016.pdf. Accessed 19 July 2020

[CR22] Cochran P, Huntington OH, Pungowiyi C, Tom S, Chapin FS, Huntington HP, Maynard NG, et al. (2013) Indigenous frameworks for observing and responding to climate change in Alaska. Climate Change and Indigenous Peoples in the United States. 10.1007/s10584-013-0735-2

[CR23] Corbett D (2016). Saĝdaĝ—to catch birds. Arct Anthropol.

[CR24] Corntassel J (2012) Re-envisioning resurgence: indigenous pathways to decolonization and sustainable self-determination. Decolon: Indig Educ Soc 1(1):86–101

[CR25] Cox S (2007) An overview of erosion, flooding, and relocation efforts in the native village of newtok. Alaska Department of Commerce, Community, and Economic Development, Division of Community Advocacy. https://www.commerce.alaska.gov/web/portals/4/pub/Newtok%20Planning%20Group/Newtok_Overview.pdf. Accessed 23 July 2019

[CR26] Curtis PD, Hauber JR (1997). Public involvement in deer management decisions: consensus versus consent. Wildl Soc Bull.

[CR27] Dannenberg AL, Frumkin H, Hess JJ, Ebi KL (2019). Managed retreat as a strategy for climate change adaptation in small communities: public health implications. Clim Change.

[CR28] DiBiase RA, Whipple KX (2011) The influence of erosion thresholds and runoff variability on the relationships among topography, climate, and erosion rate. J Geophys Res: Earth Surf 116(F4). 10.1029/2011JF002095

[CR29] Dinero SC (2013). Indigenous perspectives of climate change and its effects upon subsistence activities in the Arctic: the case of the Nets’aii Gwich’in. GeoJournal.

[CR31] Eldridge K (2016) An analysis of Archaeofauna recovered from a russian period camp on St. Paul Island, Pribilof Islands, Alaska. Arctic Anthropology. 10.3368/aa.53.2.33

[CR32] Environmental Protection Agency (EPA) (2016) Climate Change, Health, and Environmental Justice. https://www.cmu.edu/steinbrenner/EPA%20Factsheets/ej-health-climate-change.pdf Accessed 06 July 2020

[CR33] Environmental Protection Agency (EPA) (2017) Climate impacts in Alaska. https://archive.epa.gov/epa/climate-impacts/climate-impacts-alaska.html. Accessed 16 Aug 2019

[CR34] Fabricius C, Scholes R, Cundill G, Reid WV, Berkes F, Wilbanks TJ, Capistrano D (2006). Mobilizing knowledge for integrated ecosystem assessments. Bridging scales and knowledge systems: concepts and applications in ecosystem assessment.

[CR35] Foote DC, Fisher V, Rogers GW (1968). St. Paul Community Study: an economic and social analysis of St. Paul, Pribilof Islands, Alaska.

[CR36] Ford JD (2012). Indigenous health and climate change. Am J Public Health.

[CR37] Ford JD, Smit B, Wandel J (2006). Vulnerability to climate change in the Arctic: a case study from Arctic Bay, Canada. Glob Environ Change.

[CR38] Gibbs AE, Richmond BM (2017) National assessment of shoreline change—summary statistics for updated vector shorelines and associated shoreline change data for the north coast of Alaska, U.S.—Canadian border to Icy Cape. US Geological Survey Open-File Report 2017. 1107:21. 10.3133/ofr20171107

[CR39] Graham RW, Belmecheri S, Choy K, Culleton BJ, Davies LJ, Froese D, Heintzman PD, et al. (2016) Timing and causes of mid-Holocene mammoth extinction on St. Paul Island, Alaska. Proc Natl Acad Sci USA. 10.1073/pnas.1604903113 10.1073/pnas.1604903113 PMC4995940 27482085

[CR40] Herman-Mercer NM, Matkin E, Laituri MJ, Toohey RC, Massey M, Elder K,… & Mutter EA (2016). Changing times, changing stories: generational differences in climate change perspectives from four remote indigenous communities in Subarctic Alaska. Ecology and Society 21(3). 10.5751/ES-08463-210328

[CR41] Hosen N, Nakamura H, Hamzah A (2020). Adaptation to climate change: does traditional ecological knowledge hold the key?. Sustainability.

[CR42] Huntington HP (1998) Observations on the utility of the semi-directive interview for documenting traditional ecological knowledge. Artic 51(3):237–242. www.jstor.org/stable/40512135

[CR43] Huntington HP, Carey M, Apok C, Forbes BC, Fox S, Holm LK, Ivanova (2019). Climate change in context: putting people first in the Arctic. Reg Environ Change.

[CR44] Huntington OH, Watson A (2012) Interdisciplinarity, native resilience, and how the riddles can teach wildlife law in an era of rapid climate change Wicazo Sa Rev 27(2):49–73. https://www.jstor.org/stable/10.5749/wicazosareview.27.2.0049

[CR45] Ignatowski JA, Rosales J (2013). Identifying the exposure of two subsistence villages in Alaska to climate change using traditional ecological knowledge. Climatic Change.

[CR47] Intergovernmental Panel on Climate Change (IPCC) (2014) Climate change 2014: Synthesis report. https://www.ipcc.ch/site/assets/uploads/2018/02/SYR_AR5_FINAL_full.pdf. Accessed 18 July 2019

[CR48] Islam N, Winkel J (2017) Climate change and social inequality. https://www.un.org/esa/desa/papers/2017/wp152_2017.pdf Accessed 06 July 2020

[CR49] Johnson N, Behe C, Danielsen F, Krümmel E-M, Nickels S, Pulsifer PL (2016). Community-based monitoring and indigenous knowledge in a changing Arctic.

[CR50] Kim G (2020) After moving to new village, Mertarvik residents say they are living healthier, more traditional lives. https://www.alaskapublic.org/2020/07/28/after-moving-to-new-village-mertarvik-residents-say-they-are-living-healthier-more-traditional-lives/. Accessed 25 Aug 2020

[CR51] Klenk N, Meehan K (2015). Climate change and transdisciplinary science: problematizing the integration imperative. Environ Sci Policy.

[CR52] Krupnik I, Jolly D (2002). The earth is faster now indigenous observations of Artic environmental change.

[CR54] Loring PA, Gerlach SC, Penn HJ (2016). “Community work” in a climate of adaptation: responding to change in rural Alaska. Hum Ecol.

[CR55] Manrique DR, Corral S, Pereira G (2018). Climate-related displacements of coastal communities in the Arctic: engaging traditional knowledge in adaptation strategies and policies. Environ Sci Policy.

[CR56] Marin A, Berkes F (2013). Local people’s accounts of climate change: to what extent are they influenced by the media?. Wiley Interdiscip Rev: Clim Change.

[CR57] Marino E (2018). Adaptation privilege and voluntary buyouts: Perspectives on ethnocentrism in sea level rise relocation and retreat policies in the US. Glob Environ Change.

[CR58] Masterson VA, Stedman RC, Enqvist J, Tengö M, Giusti M, Wahl D, Svedin U (2017). The contribution of sense of place to social-ecological systems research: a review and research agenda. Ecol Soc.

[CR59] McCartney A, Damas David (1984). Prehistory of the Aleutian region. Handbook of North American Indians: Arctic.

[CR60] McCunn LJ, Gifford R (2018). Spatial navigation and place imageability in sense of place. Cities.

[CR61] Melvin AM, Larsen P, Boehlert B, Neumann JE, Chinowsky P, Espinet X, Martinich J (2017). Climate change damages to Alaska public infrastructure and the economics of proactive adaptation. Proc Natl Acad Sci USA.

[CR62] Moller H, Berkes F, Lyver PO, Kislalioglu M (2004). Combining science and traditional ecological knowledge: monitoring populations for co-management. Ecol Soc.

[CR63] Mugambiwa SS, Rukema JR (2019). Rethinking indigenous climate governance through climate change and variability discourse by a Zimbabwean rural community. Int J Clim Change Strateg Manag.

[CR64] Nearing MA, Govers G, Norton LD (1999) Variability in soil erosion data from replicated plots. Soil Sci Soc Am J. 10.2136/sssaj1999.6361829x

[CR65] Overland JE, Wang M, Ballinger TJ (2018). Recent increased warming of the Alaskan marine Arctic due to midlatitude linkages. Adv Atmos Sci.

[CR66] Pearce TD, Ford JD, Laidler GJ, Smit B, Duerden F, Allarut M, Andrachuk M (2009). Community collaboration and climate change research in the Canadian Arctic. Polar Res.

[CR67] Pearce T, Ford J, Willox AC, Smit B (2015) Inuit traditional ecological knowledge (TEK), subsistence hunting and adaptation to climate change in the Canadian Arctic. Arctic 233–245. 10.14430/arctic4475

[CR68] Petheram L, Stacey N, Fleming A (2014). Future sea changes: indigenous women’s preferences for adaptation to climate change on South Goulburn Island Northern Territory (Australia). Clim Dev.

[CR69] Prasad DH, Kumar ND (2014). Coastal erosion studies—a review. Int J Geosci.

[CR70] Radosavljevic B, Lantuit H, Pollard W, Overduin P, Couture N, Sachs T, Helm V, Fritz M (2016). Erosion and flooding—threats to coastal infrastructure in the Arctic: a case study from Herschel Island, Yukon Territory, Canada. Estuaries Coasts.

[CR71] Raynolds MK, Walker DA, Ambrosius KJ, Brown J, Everette KR, Kanevskiy M, Kofinas GP (2013). Cumulative geoecological effects of 62 years of infrastructure and climate change in ice-rich permafrost landscapes, Prudhoe Bay Oilfield, Alaska. Glob Change Biol.

[CR72] Richards G, Frehs J, Myers E, Van Bibber M (2019). The Climate Change and Health Adaptation Program: indigenous climate leaders’ championing adaptation efforts. Health Promot Chronic Dis Prev Can: Res Policy Pract.

[CR73] Rosales J, Chapman JL (2015). Perceptions of obvious and disruptive climate change: community-based risk assessment for two native villages in Alaska. Climate.

[CR74] Rossi M (2019). Surrendering to the sea by choice. Nat Clim Change.

[CR75] Rubicz RC (2007) Evolutionary consequences of recently founded Aleut communities in the Commander and Pribilof Islands. Ph.D. Thesis, University of Kansas, p 178. Lawrence, Kansas, USA

[CR76] Salick J, Ross N (2009). Traditional peoples and climate change. Glob Environ Change.

[CR77] Sanò M, Jiménez JA, Medina R, Stanica A, Sanchez-Arcilla A, Trumbic I (2011). The role of coastal setbacks in the context of coastal erosion and climate change. Ocean Coast Manag.

[CR78] Shen S, Ristroph EB (2020). The relationship between climate vulnerability and disaster declarations: a case study of flood-prone indigenous communities in Alaska. Nat Hazards Rev.

[CR79] Smith N, Sattineni A (2016). Effect of erosion in Alaskan coastal villages.

[CR80] Snyder R, Williams D, Peterson G (2003) Culture loss and sense of place in resource valuation: economics, anthropology, and indigenous cultures. In: Jentoft S, Minde H, Nilsonp R (ed.) Indigenous peoples: Resource management and global rights. Eburon, Utrecht, Netherlands, pp 107–123

[CR81] TDX (2019) TDX mission, history, about us. https://www.tdxcorp.com/. Accessed 23 Aug 2019

[CR82] Thomas DR (2003). A general inductive approach for qualitative data analysis.

[CR83] Thomson J, Rogers EW (2014) Swell and sea in the emerging Arctic Ocean. Geophysical Research Letters. 10.1002/2014GL059983

[CR84] Torrey BB (1983) Slaves of the harvest. Tanadgusix Corporation, Anchorage, Alaska, USA

[CR85] Towell R, Ream RR, Bengtson J, Williams M, Sterling J (2016) 2016 northern fur seal pup production and adult male counts on the Pribilof Islands, Alaska. Memorandum for the Record, November 29, 2016. Available AFSC, Marine Mammal Laboratory, NOAA, NMFS 7600 Sand Point Way NE, Seattle WA

[CR86] Tribal Adaptation Menu Team (2019). Dibaginjigaadeg Anishinaabe Ezhitwaad: a tribal climate adaptation menu.

[CR87] United States Census Bureau (2010) American Fact Finder. https://factfinder.census.gov/bkmk/cf/1.0/en/zip/99660/POPULATION/DECENNIAL_CNT. Accessed 18 July 2019

[CR88] United States Army Corps of Engineers (USACE) Alaska District (2006a) Alaska Village erosion technical assistance program: an examination of erosion issues in the communities of Bethel, Dillingham, Kaktovik, Kivalina, Newtok, Shishmaref, and Unalakleet. http://66.160.145.48/coms/cli/AVETA_Report.pdf. Accessed 11 Sep 2019

[CR89] United States Army Corps of Engineers (USACE) Alaska District (2006b) AVETA report summary—Shishmaref, Alaska. https://www.poa.usace.army.mil/Portals/34/docs/civilworks/BEA/Shishmaref_Final%20Report.pdf. Accessed 24 July 2019

[CR90] United States Army Corps of Engineers (USACE) Alaska District (2008) Erosion information paper—Koyukuk, Alaska. Final report. https://www.poa.usace.army.mil/Portals/34/docs/civilworks/BEA/Koyukuk_Final%20Report.pdf. Accessed 24 July 2019

[CR91] United States Code of Federal Regulations (1971). 43 U.S. Code: Alaska Native Claims Settlement Act (ANCSA). Public Law 92-203; 43 CFR 2653, 36 CFR Part 79

[CR93] United States Government Accountability Office (US GAO) (2003) Alaska native villages most are affected by flooding and erosion, but few qualify for federal assistance. https://www.gao.gov/assets/250/240810.pdf. Accessed 24 July 2019

[CR94] United States Government Accountability Office (US GAO) (2009) Alaska native villages limited progress has been made on relocating villages threatened by erosion and flooding. https://www.gao.gov/assets/300/290468.pdf. Accessed 27 May 2020

[CR95] Veniaminov I (1984). Notes on the Islands of the Unalashka District.

[CR96] Vermaire JC, Pisaric MFJ, Thienpont JR, Mustaphi CJC, Kokelj SV, Smol JP (2013) Arctic climate warming and sea ice declines lead to increased storm surge activity. Geophys Res Lett. 10.1002/grl.50191

[CR97] Walters KL, Beltran R, Huh D, Evans-Campbell T (2011) Dis-placement and dis-ease: land, place, and health among American Indians and Alaska Natives. Commun Neighb Health: Expand Bound Place. 163–199. 10.1007/978-1-4419-7482-2_10

[CR98] Wang M, Overland JE (2009) A sea ice free summer Arctic within 30 years?. Geophys Res Lett 36(7). 10.1029/2009GL037820

[CR99] Whyte KP (2014). A concern about shifting interactions between indigenous and non-indigenous parties in US climate adaptation contexts. Interdiscip Environ Rev.

[CR100] Williams P, Alessa L, Abatzoglou JT, Kliskey A, Witmer F, Lee O, Trammell J (2018). Community-based observing networks and systems in the Arctic: human perceptions of environmental change and instrument-derived data. Regional Environ Change.

[CR101] Willox AC, Harper SL, Edge VL, Landman K, Houle K, Ford JD, Rigolet Inuit Community Government (2013). The land enriches the soul: on climatic and environmental change, affect and emotional health and well-being in Rigolet, Nunatsiavut, Canada. Emot Space Soc.

[CR102] Wilson NJ (2014). The politics of adaptation: subsistence livelihoods and vulnerability to climate change in the Koyukon Athabascan village of Ruby, Alaska. Hum Ecol.

